# Xeniaphyllane and Xeniolide Diterpenes from the Deep-Sea Soft Coral *Paragorgia arborea*

**DOI:** 10.1021/acsomega.4c06361

**Published:** 2024-09-25

**Authors:** Sam Afoullouss, Ryan M. Young, Laurence K. Jennings, Jason Doyle, Karen Croke, Debora Livorsi, John H. Adams, Mark P. Johnson, Olivier P. Thomas, A. Louise Allcock

**Affiliations:** † School of Biological and Chemical Sciences, Ryan Institute, University of Galway, University Road, H91 TK33 Galway, Ireland; ‡ School of Natural Sciences, Ryan Institute, University of Galway, University Road, H91 TK33 Galway, Ireland; § Department of Chemistry, University of South Florida, 4202 E. Fowler Avenue, CHE 205, Tampa, Florida 33620, United States; ∥ Center for Global Health & Inter-disciplinary Research, College of Public Health, University of South Florida, 3720 Spectrum Boulevard, STE 404, Tampa, Florida 33612, United States

## Abstract

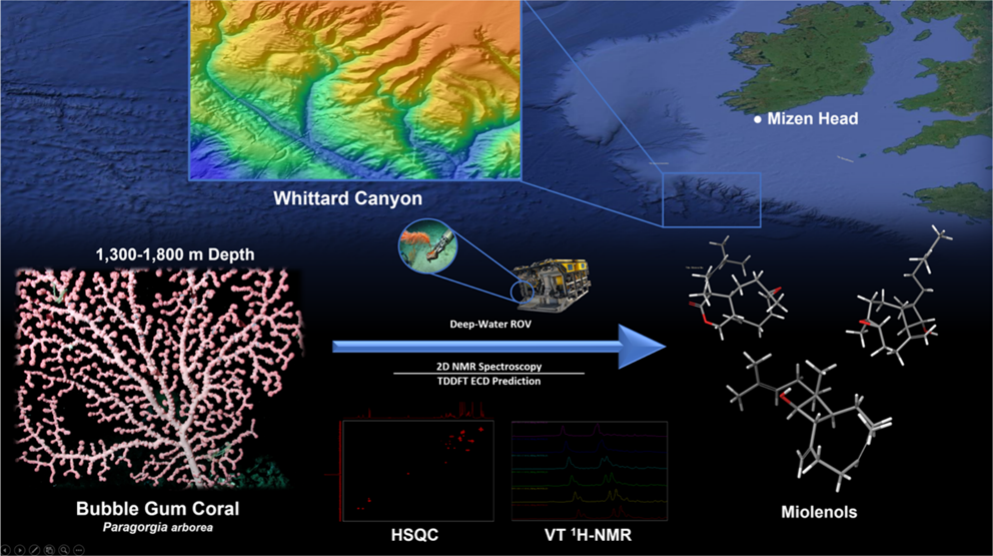

As exploration of ocean depths >1000 m is only possible by expensive remotely operated underwater vehicles, deep-sea invertebrates represent a largely untapped source of marine metabolites for potential applications in medicine. Our current study aims to investigate these deep-sea invertebrates in Ireland to discover new biological and chemical diversity. Here, we investigate the bubble gum coral, *Paragorgia arborea*, collected at 1500 m depth from Whittard canyon in the Northeastern Atlantic. This species was selected following chemical profiling and biological screening. The isolation and structure elucidation of the main metabolites yielded three new diterpenes, namely, miolenol (**1**) and epoxymiolenol (**2**) characterized by the rare bicyclo[7.2.0]undec-4-ene skeleton, and the xeniolide epoxycoraxeniolide A (**3**), together with five known diterpenes. The structures of the new compounds were identified through extensive NMR analysis with their absolute configurations assigned by comparison between experimental and TDDFT-calculated ECD. The eight compounds were screened for cytotoxicity and antimalarial activity, and none displayed noteworthy bioactivity.

## Introduction

Submarine canyons host unique and complex ecosystems including rich deep-sea reef systems.^1^ Advances in deep-sea technologies such as remotely operated underwater vehicles (ROVs) have opened the access to these underexplored reefs, revealing a unique benthic biodiversity.^2,3^ The extreme physicochemical conditions of the deep oceans characterized by high pressure, low temperatures, and absence of light, together with intense competition for food and space,^4,5^ have driven unique metabolic adaptations.^6,7^ The fauna inhabiting these reefs has then become a new horizon for marine biodiscovery and deep-sea waters could become a rich source of metabolites with original chemical scaffolds and bioactivity,^8,9^ as exemplified by the recent discovery of a novel lipoglycotripeptide named characellide A from a deep-sea sponge of the genus *Characella*.^10^

With the aim to discover new biological and chemical diversity in Irish submarine canyons and their deep-sea coral gardens, the ROV *Holland I* allowed sampling at depths down to 2500 m in the Whittard Canyon of the Northeastern Atlantic. The collection of deep-sea invertebrates included a range of octocorals well known for the production of bioactive metabolites, which frequently include terpenes and their derivatives, such as furanocembranoid diterpenes from deep-sea primnoid octocorals,^11^ briarane diterpenes from an Antarctic sea pen,^12^ and antiplasmodial cembrane-type diterpenoids named caucanolides from a deep-sea gorgonian.^13^

**Figure 1:**
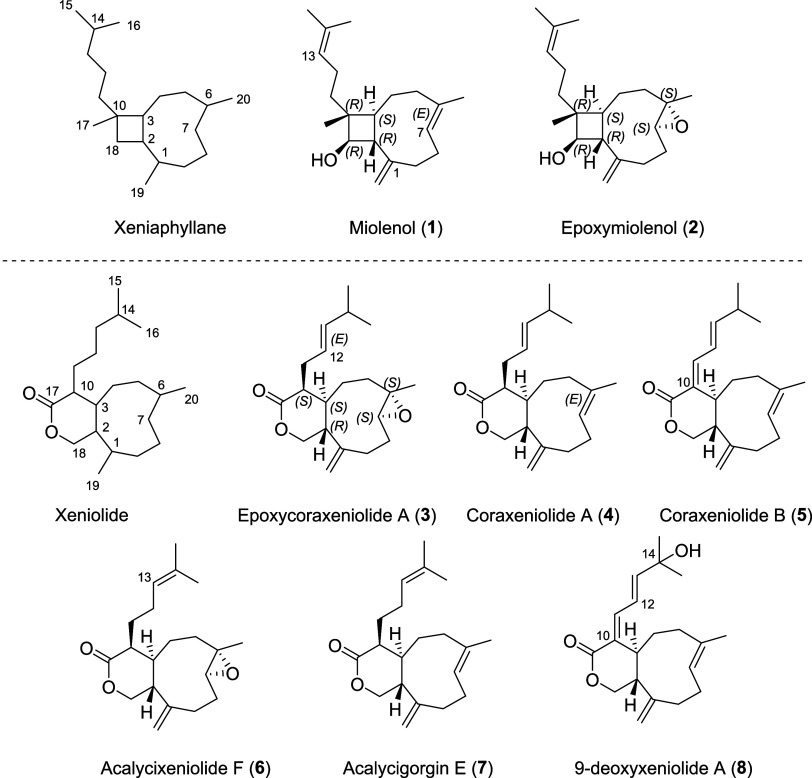
Structures of the diterpenoids isolated from the deep-sea octocoral *Paragorgia arborea*.

An extensive bioactivity screening process combined with chemical profiling led us to target the bubble gum coral *Paragorgia arborea* (Scleralcyonacea: Coralliidae) as its organic extract showed antimalarial activity and cytotoxicity. Few chemical studies have been reported on this octocoral so far, and they mainly led to the identification of xenicane diterpenoids.^14−16^ While this class of compounds appeared to be present in the organic extract of our specimens, as indicated by characteristic exomethylene and methyl signals in the ^1^H NMR spectrum, some other unique signals prompted us to undertake an in-depth chemical study of this specimen. This chemical investigation resulted in the isolation of two new xeniaphyllane diterpenoids named miolenol^1^ (**1**) and epoxymiolenol (**2**), both containing a unique cyclobutanol ring, a new xeniolide, epoxycoraxeniolide A (**3**), together with five known xeniolides, coraxeniolide A (**4**) and B (**5**),^17^ acalycixeniolide F (**6**),^18^ acalycigorgin E (**7**),^19^ and 9-deoxyxeniolide A (**8**) (Figure 1).^20^

## Results and Discussion

After being freeze-dried, the biomass was extracted thoroughly with dichloromethane in a Soxhlet apparatus in the search for terpenoids. The extract was fractionated by reversed-phase *vacuum* liquid chromatography on C_18_ with solvents of decreasing polarities from H_2_O, to MeOH and then CH_2_Cl_2_. The methanolic fraction was then subject to successive reversed-phase HPLC purification yielding two new xeniaphyllane-type diterpenes (**1** and **2**), along with one new (**3**) and five known (**4**–**8**) xeniolide-type diterpenes.

Compound **1** was isolated as a colorless oil, and its molecular formula was determined as C_20_H_32_O, based on the protonated molecule [M + H]^+^ at *m*/*z* 289.2536 in the (+)-ESI-TOFMS spectrum. The ^1^H NMR spectrum evidenced the presence of two conformers for **1**, and we started elucidating the structure of the major conformer named **1a**. The presence of a minor conformer **1b** rather than a diastereoisomer was determined through chemical exchange correlations observed in the NOESY spectrum, and by variable-temperature ^1^H NMR as explained later. Examination of the HSQC and HMBC spectra in CDCl_3_ established the presence of four methyl groups with signals at δ_H_ 1.69 (s, H_3_-15), δ_C_ 25.9 (C-15); δ_H_ 1.62 (s, H_3_-16), δ_C_ 17.8 (C-16); δ_H_ 1.56 (s, H_3_-20), δ_C_ 16.7 (C-20); δ_H_ 0.95 (s, H_3_-17), and δ_C_ 13.2 (C-17); an exocyclic methylene with signals at δ_H_ 4.90 (br s, H-19a), 4.87 (br s, H-19b), δ_C_ 114.2 (C-19), and δ_C_ 150.6 (C-1); a trisubstituted double bond with signals at δ_H_ 5.27 (dd, *J* = 11.0, 5.0 Hz, H-7), δ_C_ 125.0 (C-7), and δ_C_ 134.8 (C-6); and a second one with signals at δ_H_ 5.12 (t, *J* = 6.0 Hz, H-13), δ_C_ 124.8 (C-13), and δ_C_ 131.7 (C-14) (Table 1). These double bonds accounted for three hydrogen deficiencies, indicating that the remaining two should be due to rings. The oxygen was determined to be in the form of a secondary alcohol with signals at δ_H_ 3.52 (br d, *J* = 8.0 Hz, H-18) and δ_C_ 75.8 (C-18), supported by a COSY correlation between H-18 and the exchangeable proton at δ_H_ 1.53.

**Table 1:** NMR Spectroscopic Data (600 MHz) for Miolenol (**1**), Epoxymiolenol (**2**), and Epoxycoraxeniolide A (**3**) in CDCl_3_

	miolenol (**1**)							
	major (**1a**)		minor (**1b**)		epoxymiolenol (**2**)		epoxycoraxeniolide A (**3**)	
pos	δ_C_, type	δ_H_ mult. (*J* in Hz)	δ_C_, type	δ_H_ mult. (*J* in Hz)	δ_C_, type	δ_H_ mult. (*J* in Hz)	δ_C_, type	δ_H_ mult. (*J* in Hz)
1	150.6, C		n.o.		148.4, C		150.8, C	
2β	60.2, CH	2.19 t (9.0)	60.1, CH	2.06 m	59.4, CH	2.44 t (6.5)	48.5, CH	2.37 dd (6.0, 4.0)
3α	43.1, CH	1.38 m	47.7, CH	1.03 m	40.4, CH	1.47 m	43.9, CH	2.16 m
4α	29.0, CH_2_	1.64 m	31.7, CH_2_	1.69 m	27.2, CH_2_	1.80 br d (12.0)	28.2, CH_2_	1.81 br d (14.0)
4β		1.39 m				1.42 m		1.09 dt (14.0, 7.0)
5α	39.7, CH_2_	1.90 m	35.0, CH_2_	1.57 m	38.5, CH_2_	0.98 m	39.7, CH_2_	0.95 m
5β		2.10 m		2.51 m		2.10 br d (12.0)		2.24 m
6	134.8, C		n.o.		59.6, C		59.2, C	
7	125.0, CH	5.27 dd (11.0, 5.0)	130.2, CH	5.35 m	64.3, CH	2.83 br d (10.0)	61.9, CH	2.88 dd (6.0, 5.0)
8α	29.9, CH_2_	2.06 m	30.0, CH_2_	2.46 m	30.7, CH_2_	2.27 m	25.2, CH_2_	2.28 m
8β		2.35 m		2.13 m		1.32 m		1.47 dt (13.0; 6.0)
9α	33.7, CH_2_	2.05 m	40.8, CH_2_	2.48 m	28.5, CH_2_	2.15 t (8.0)	33.7, CH_2_	2.12 m
9β		2.17 m		1.95 m		2.32 m		2.56 m
10	43.4, C		n.o.		44.6, C	-	43.0, CH	2.84 dt (8.0, 6.0)
11a	42.7, CH_2_	1.42 m	43.1, CH	1.39 m	42.3, CH_2_	1.41 m	29.9, CH_2_	2.57 m
11b								2.16 m
12a	23.2, CH_2_	2.00 m	23.2, CH_2_	2.00 m	23.0, CH_2_	2.01 m	122.9, CH	5.29 ddd (15.0, 9.0, 5.0)
12b		1.96 m		1.96 m		1.96 m		
13	124.8, CH	5.12 t (6.0)	124.8, CH	5.12 m	124.9, CH	5.10 t (7.0)	141.2, CH_2_	5.47 dd (15.0, 7.0)
14	131.7, C		n.o.		131.6, C		31.3, CH	2.25 m
15	25.9, CH_3_	1.69 s	25.9, CH_3_	1.69 s	25.7, CH_3_	1.69 s	22.7, CH_3_	0.95 d (6.5)
16	17.8, CH_3_	1.62 s	17.9, CH_3_	1.62 s	17.7, CH_3_	1.62 s		
17	13.2, CH_3_	0.95 s	12.8, CH_3_	0.93 s	12.5, CH_3_	0.98 s	174.7, C	
18α	75.8, CH	3.52 br d (9.0)	79.1, CH	3.65, d (9.0)	75.1, CH	3.52 br t (6.5)	70.9, CH_2_	3.96 t (12.0)
18β								4.28 dd (12.0, 6.5)
19a	114.2, CH_2_	4.90 br s	111.2, CH_2_	4.90 br s	114.5, CH_2_	4.99 br s	113.9, CH_2_	5.17 br s
19b		4.87 br s		4.87 br s		4.89 br s		5.06 br s
20	16.7, CH_3_	1.56 s	22.1, CH_3_	1.56 s	17.2, CH_3_	1.15 s	16.3, CH_2_	1.37 s

**Figure 2:**
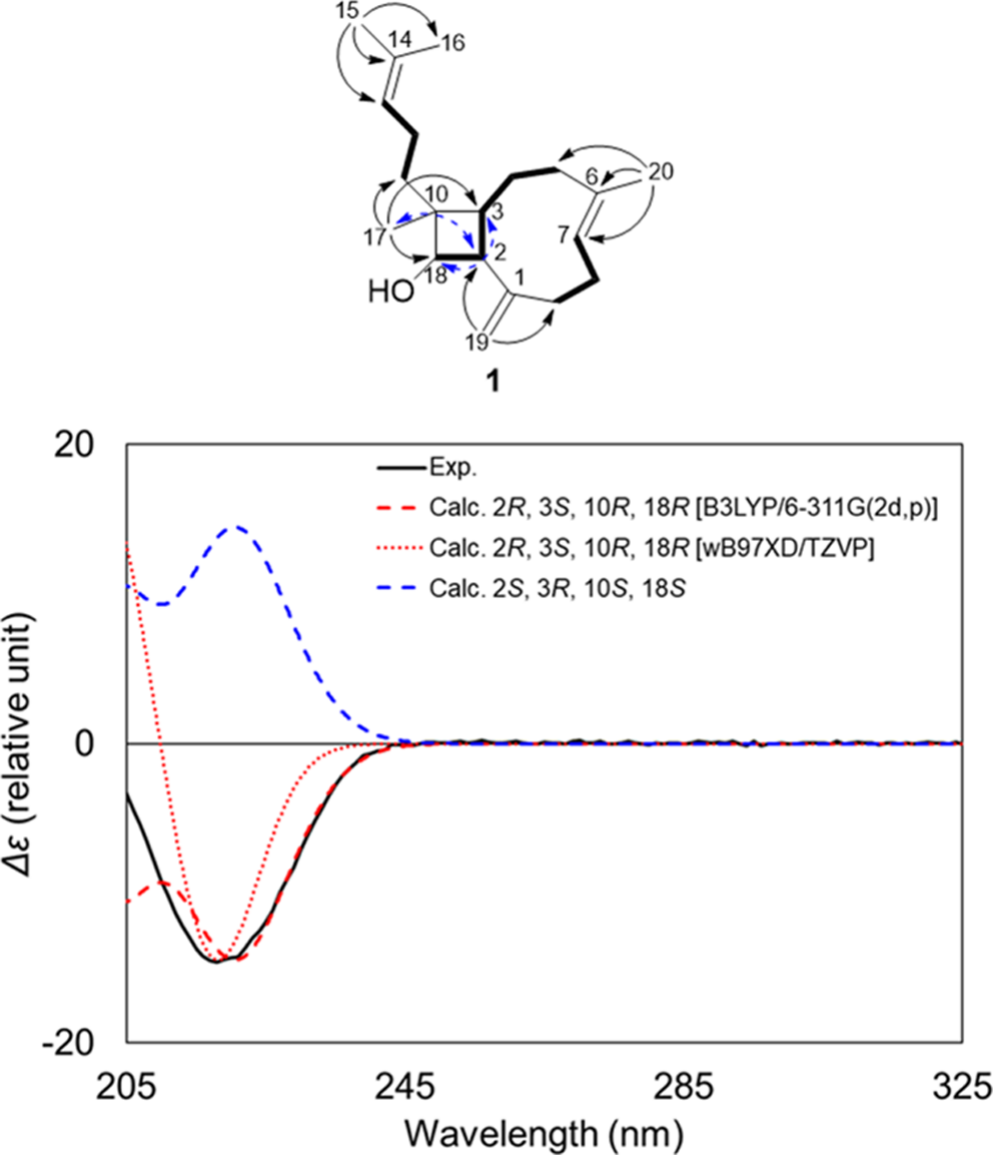
Key COSY (bold), HMBC (black arrows), and NOESY (dashed blue arrows) correlations for miolenol (**1**) along with the calculated and experimental ECD spectra for **1**.

Using the COSY spectrum, three spin systems were evidenced starting from the secondary alcohol H-18, and the olefinic protons H-7 and H-13 (Figure 2). HMBC correlations from the H_3_-15 and H_3_-16 olefinic methyls to C-13 and C-14 allowed an isoprenyl group to be assigned. The HMBC correlations from the H_3_-20 methyl allowed the assignment of a trisubstituted olefin connecting the H-7 and H-18 spin systems. The key H_2_-19/C-2/C-1 and C-9 HMBC correlations allowed another exomethylene connection between the two spin systems, leading to the assignment of a nonene ring, characteristic of xenicane-type terpenes. The last methyl at δ_H_ 0.95 (s, H_3_-17) was placed in a vicinal position with C-18 through a key H_3_-17/C-18 HMBC correlation. H_3_-17 also displayed a single broad HMBC correlation to the three carbons at C-3, C-10, and C-11 with very close chemical shifts. Further HMBC correlations from H_2_-11 and H-3 to C-17 then confirmed the linkage of the final two partial structures to form a cyclobutanol ring. Therefore, we could conclude that miolenol (**1**) belonged to the family of xeniaphyllane diterpenes characterized by a bicylo[7.2.0]undecane. The name xeniaphyllane was proposed in 1978 based on the isolation of the first member of this diterpene family in a soft coral of the genus *Xenia* and also the analogy with caryophyllane, a family of sesquiterpenes found in plants as well as soft corals that also possess this bicyclic system.^21^ Despite a biosynthetic pathway closely related to the previously reported xenicane (first named xeniane), the numbering of the carbon chain was proposed to differ. Later, another numbering was also adopted, and we decided to follow this numbering.^22,23^

The relative configurations of the four stereogenic centers of the cyclobutane ring of **1** were investigated by using a NOESY experiment. Like for most of the members of this family, the C-6/C-7 olefin was established as *E* based on the NOESY correlation between H_2_-8 and H_3_-20. The relative configuration of the cyclobutanol ring of **1** was assigned from the key NOEs of H_3_-17 with H-4β, and H-2 requiring these protons to occupy the same β face of the molecule. Then, the key NOEs of H-18 with H_2_-11 and H-3 placed these protons on the opposite α face. These orientations were consistent with the coupling constant value of the signal at δ_H_ 2.19 (t, *J* = 9.0 Hz, H-2).

Finally, the absolute configuration of **1** was determined by a comparison between the experimental and calculated electronic circular dichroism (ECD) spectra. Due to the flexibility of the molecule, the minimum energy conformer search was constrained using experimental NOESY correlations and coupling constant values. Time-dependent density functional theory (TDDFT) was then used to calculate ECD spectra of the 42 resulting conformers at the M06-2X/def2-TZVP//B3LYP/6-311G(2d,p) level of theory. The calculated ECD spectrum for the absolute configuration 2*R*, 3*S*, 10*R*, and 18*R* matched the experimental spectrum (Figure 2). Due to the limited number of stereochemical studies on this family and in order to allow a more reliable assignment, the ECD of the optimized conformers of **1** were calculated again using another functional/basis set combination (ωB97XD/TZVP). These data matched the previous calculations further supporting our assignment. Surprisingly, the absolute configuration for most of the coral-derived xeniaphyllane family has not been determined, and while previous structures contained stereo assignments, these were to indicate the relative and not absolute configuration. However, while undetermined, these assignments have been incorrectly reported as absolute configurations in numerous databases and reviews.^23^ More recently, several other soft coral caryophyllene-type terpenes have reported the same absolute configuration as reported here. The TDDFT-calculated ECD of sinuhirfuranone A, isolated from the soft coral *Sinularia hirta*, led to the assignment of the same absolute configuration.^24^ The synthesis of the similar diterpene antheliolide A established the stereochemical revision of the four-membered ring to the same absolute configuration as reported here.^25^ Furthermore, the configuration of coral-derived caryophyllene-type sesquiterpenes, nanonorcaryophyllenes, was opposite to those based on optical rotation data from plant studies.^26^ Along with the data we present here, these recent stereochemical studies suggest that the unassigned configurations of earlier reported xeniaphyllanes should be assigned as enantiomers to the plant-derived (−)-caryophyllene, and this class likely needs a full investigation and correction.^23^

**Figure 3:**
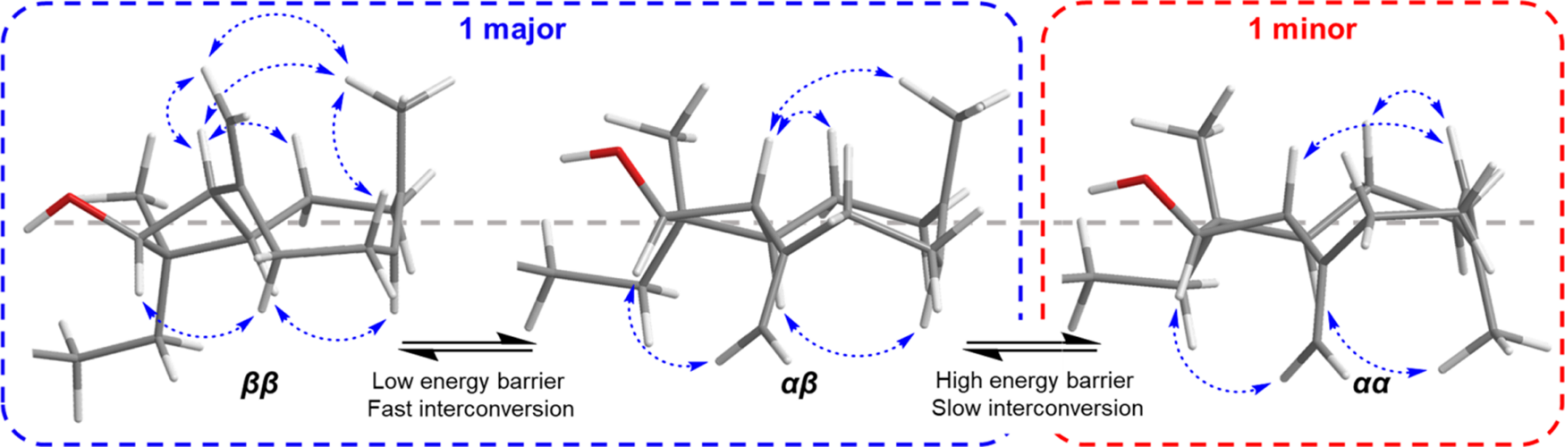
Geometries of the three interconverting conformers of miolenol A (**1**) with NOESY correlations indicated for the conformer assignment. The side chain was cropped from the image.

Initial examination of the ^1^H NMR spectrum of miolenol (**1**) showed the presence of what appeared to be a doubling of signals in a 3:1 ratio. Exchange correlations observed in the NOESY spectrum (Figures S10–S17) rather suggested that the interconversion between two conformers **1a** (major) and **1b** (minor) was responsible for the observed effect, similar to those reported for the protoxenicins.^27^ Conformational interconversion was confirmed by a variable-temperature ^1^H NMR experiment (Figure S12), obtained in deuterated pyridine at 10 °C increments from 15 to 65 °C. These experiments demonstrated that the proton signals of **1a** at δ_H_ 1.61 (H_3_-15) and δ_H_ 1.63 (H-15) for **1b** at 15 °C were merging at δ_H_ 1.66 above 65 °C. An in-depth literature review of bicyclo[7.2.0]undecene molecular dynamics revealed some data showing the presence of three interconverting conformers for (−)-β-caryophyllene named αα (48%), βα (28%), and ββ (24%). Conformers αα and βα interconvert rapidly, producing the major NMR signals at room temperature, while there is slow interconversion to ββ due to a higher energy barrier (Δ*H* = 14.7 kcal/mol) leading to the minor NMR signals observed.^28^ We hypothesize that a similar phenomenon occurs in miolenol (**1**). NOE correlations from H_2_-19 to H-2 and H-18 as well as from H-18 to H-9α in **1a** indicated it is rapidly converting between ββ and αβ, which can only be observed at lower-temperature NMR spectra. While the NOE correlations of **1b** suggested that it was in an αα conformation (Figure 3), no correlations were observed that indicated the presence of the βα conformer. This data for the newly isolated miolenol (**1**) is in agreement with the previous work on caryophyllene, with matching enantiomeric conformers observed along with matching ratios based on ^1^H NMR integration (76% ββ/αβ and 24% αα).^28^

**Figure 4:**
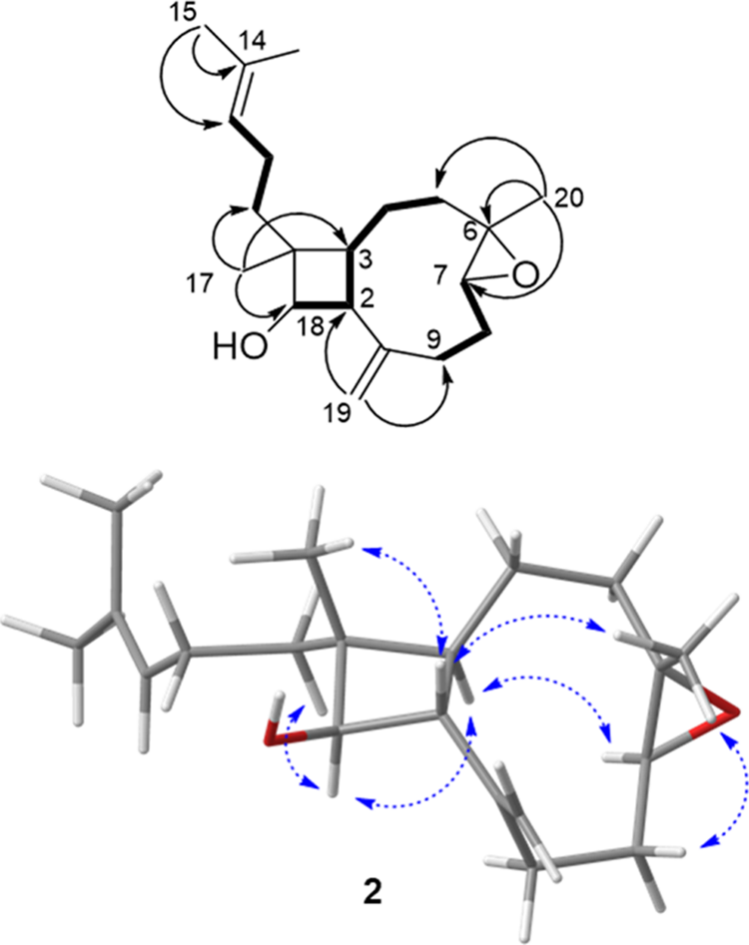
Key COSY and HMBC correlations establishing the planar structures of epoxymiolenol (**2**); key NOEs (blue arrows) for **2**.

Compound **2** was isolated as a colorless oil, and its molecular formula was determined as C_20_H_32_O_2_, based on the (+)-ESI-TOFMS spectrum ([M + H]^+^ at *m*/*z* 305.2472). Comparison of 1D and 2D NMR spectra of **1** and **2** revealed that **2** also belongs to the xeniaphyllane family, differing from miolenol (**1**) by a lack of an olefin and two new oxygenated carbons at δ_C_ 59.6 (C-6) and δ_C_ 64.3 (C-7) consistent with the epoxidation of the double bond of **1** at C-6/C-7. The trisubstituted epoxide was confirmed by a deshielded signal at δ_H_ 2.83 (br d, *J* = 10.0 Hz, H-7) and HMBC correlations from H_3_-20 to C-5/C-6/C-7. Once the planar structure of **2** was established, the relative configuration of the cyclobutanol ring was found to be the same as **1** due to very similar chemical shifts of the corresponding signals especially for the ^13^C NMR signals. Even though the ^3^*J* coupling constants from H-2 with values of 6.5 Hz are lower than expected for *trans* relative configurations, we assume this change is due to a change of conformation following the epoxidation of the double bond. The absence of another low-energy minor rotamer for **2** is proof of the change in its conformational profile. The relative configuration of the epoxide group of **2** stemming from the *E* double bond of **1** was deduced from the key H-3/H-7 and H-2/H_3_-20 NOE correlations (Figure 4). Then, the assessment of the relative configuration of the epoxide with respect to the cyclobutanol required a comparison with literature data. Through conformational analysis of the cyclononene analogues, it is largely accepted that epoxidation results in the epoxide being in a pseudoequatorial position relative to the nonane ring, with the epoxide proton and methyl in an anticonfiguration.^29^ The absolute configuration of epoxymiolenol (**2**) was finally determined by comparison of the experimental and computational ECD spectra, using the same methodology as above, and the experimental spectrum perfectly matched the calculated spectrum of the (2*R*, 3*S*, 6*S*, 7*S*, 10*R*, 18*R*) enantiomer (Figure S49).

**Figure 5:**
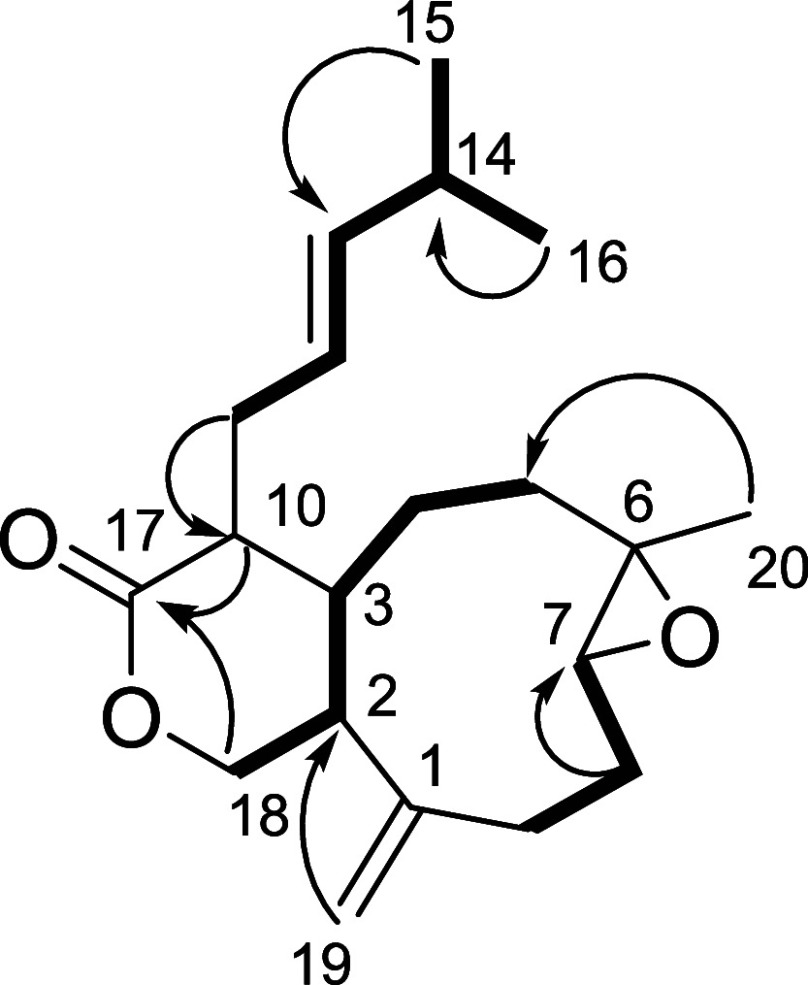
Key COSY and HMBC correlations establishing the planar structures of epoxycoraxenolide A (**3**).

Compound **3** was isolated as a pale-yellow oil. Its molecular formula was determined by (+)-ESI-TOFMS as C_20_H_30_O_3_ (*m*/*z* = 319.2269 for [M + H]^+^). Interpretation of ^1^H NMR data revealed the presence of an isopropyl group with methyl signals at δ_H_ 0.95 (d, *J* = 6.5; H_3_-15/16), δ_C_ 22.7 (C-15/C-16), a third methyl group with signals at δ_H_ 1.37 (s, H_3_-20) and δ_C_ 16.3 (C-20), and an exocyclic methylene group at δ_H_ 5.17 (br s, H-19a), 5.06 (br s, H-19b) and δ_C_ 113.9 (C-19). Comparison of the HSQC spectra of epoxymiolenol (**2**) and **3** indicated the shared presence of a C-6/C-7 epoxide-bearing cyclononane ring but also the absence of the cyclobutanol. An additional disubstituted double bond of the *E* configuration was deduced from two signals at δ_H_ 5.47 (dd, *J* = 15.0, 7.0, H-13) and δ_H_ 5.29 (ddd, *J* = 15.0, 9.0, 5.0 Hz, H-12) which was easily located on the side chain through COSY correlations from H_3_-15/H_3_-16. Interpretation of the signal at δ_C_ 174.7 (C-17) suggested the presence of an ester functional group. By comparison with other diterpenes isolated with xeniaphyllanes, we then assumed that compound **3** was a xeniolide. The characteristic signals of two ABX systems at δ_H_ 4.28 (dd, *J* = 12.0, 7.0 Hz, H-18β) and δ_H_ 3.96 (t, *J* = 12.0 Hz, H-18α) came as a confirmation of this assumption (Figure 5).

Comparison with data of reported xeniolides led us to propose that **3** was an epoxy derivative of the known coraxeniolide A (**4**) at C-6/C-7.^17,30^ Through comparison of NMR data, the relative configuration of epoxycoraxeniolide A (**3**) was found to be identical to that of coraxeniolide A (**4**). Further key H-3/H-7 and H-2/H_3_-20 NOESY correlations also indicated the same configuration of the new epoxide group as that of **2**. The absolute configuration (2*R*, 3*S*, 6*S*, 7*S*, 10*S*) for epoxycoraxeniolide A (**3**) was determined by comparison of experimental and calculated ECD spectra (Figure S50) using the same method as for miolenols.

Interestingly, the co-occurence of xeniaphyllane and xeniolide diterpenes has already been observed in shallow water soft corals of the genus *Xenia*, but this is the first report from a deep-sea soft coral.^29^ This observation shows that both families of diterpenes are not restricted to deep-sea organisms but are distributed in diverse families of octocorals from shallow water. The presence of both families of diterpenes in the same specimen also confirms that their metabolic pathways are closely related, therefore justifying the common numbering used. The cyclobutanol ring of miolenols could correspond to a missing piece in the metabolic pathway of xenicanes preceding the cleavage of the C-18/C-10 bond that would lead to the lactone ring of xeniolides.^22^

**Figure 6:**
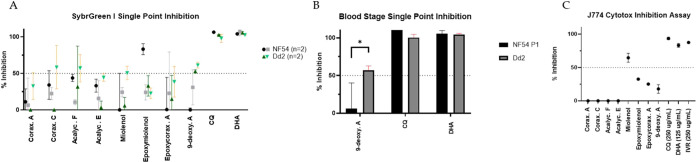
Xenicanes evaluated for inhibition against *Plasmodium falciparum* strains NF54 (drug-sensitive), Dd2 (drug-resistant) (5 μg mL^–1^), and J774 macrophage toxicity (10 μg mL^–1^). (A) Error bars represent means with SD for technical replicates (*i* = 2) with experimental replicates shown by independent points (*i* = 2). (B) Two-way ANOVA of the combined data between NF54 and Dd2 to 9-deoxyxeniolide A (**8**) (*P* = 0.0113). (C) Cytotoxicity against J774 macrophages.

Diterpenoids **1**–**8** were screened for antiplasmodial activity against *Plasmodium falciparum* strains NF54 (drug-sensitive) and Dd2 (drug-resistant) at a concentration of 5 μg mL^–1^. Cytotoxicity was assessed against J774 macrophage cell lines at concentrations of 10 μg mL^–1^. Results from both strains indicate modest antiplasmodial activity for all metabolites (Figure 6). 9-Deoxyxeniolide A (**8**) showed >50% activity (57%) against the Dd2 strain in blood stage. Only miolenol (**1**) showed cytotoxicity with 65% inhibition against J744 macrophages.

## Experimental Information

### General Experimental Procedures

Optical rotations were measured on a UNIPOL 1000 Polarimeter. UV and ECD data were measured on a Chirascan V100 instrument with a 1.0 cm quartz cuvette (Applied Photophysics, Leatherhead, U.K.). NMR experiments were obtained on a 600 MHz Varian spectrometer equipped with a cryoprobe. Chemical shifts (δ in ppm) are referenced using residual peak of CDCl_3_ (δ_H_ 7.26 for ^1^H and δ_C_ 77.16 for ^13^C) and pyridine-*d*_5_ (δ_H_ 8.74, 7.58, 7.22 and δ_C_ 150.35, 135.91, 123.87). High-resolution electrospray ionization mass spectrometry (HRESIMS) data were obtained from a Q-ToF Agilent 6540 instrument in positive mode. Preparative HPLC was performed using a Jasco PU-2087 Plus equipped with a UV 2075 Plus detector and using an Agilent 1260 analytical HPLC series equipped with a DAD detector.

### Animal Material

Two specimens were collected close together using the remotely operated vehicle Holland I during expedition CE17008 of RV Celtic Explorer at approximately 1,500 m depth and at 48.830°N, 11.048°W. All epibionts were removed from the surface of the corals, and a small amount was retained in 96% ethanol as a voucher specimen for DNA analysis (SFI081) and morphological identification (SFI070). The rest of the sample was lyophilized and stored at −20 °C. Based on colony morphology and DNA sequencing of the octocoral mitochondrial gene MutS (GenBank Accession No. OM240806), the specimen was identified as *Paragorgia arborea*.

### Extraction and Purification

A wet biomass (758 g) was lyophilized and extracted with 1 L of dichloromethane utilizing a Soxhlet, resulting in 7.2 g of extract. Fractionation of the extract was achieved using reversed-phase *vacuum* liquid chromatography on C_18_, giving seven fractions (Figure S2). The methanolic fraction (675 mg) was purified by RP-HPLC-DAD on a semipreparative phenylhexyl column of 5 μm, 10 × 250 mm^2^ (Xselect, Waters). Mobile phase consisted of A (H_2_O) and B (CH_3_CN), at a flow rate of 5 mL/min. The method was developed on 60 min acquisition time: isocratic 30% B for 4 min, then linear gradient to 70% B at 45 min, raised to 100% B at 47 min, held at 100% B until 54 min, back to 30% B in 1 min, and held at that percentage of B for 5 min. Initial purification, using acidified solvents (0.1% TFA), appeared to cause degradation of metabolites after drying, indicated by ^1^H NMR experiments (Figure S3), promoting the use of nonacidic solvent for subsequent purifications. Subfraction 10 (t_R_ 34 min, 21 mg) containing miolenol A (**1**) underwent further purification, conducted on an analytical T3 column 4.6 × 250 mm 5 μm (Xselect, Waters), using isocratic condition at 85% B for 31 min, yielding miolenol A (t_R_ 16 min, 0.66 mg, 9.2 × 10^–5^% w/w). Subfraction 3 (*t*_R_ 22 min, 18 mg) containing miolenol B (**2**), was repurified on an analytical C_18_ column 4.6 × 250 mm^2^ 5 μm (Xselect, Waters) (*t*_R_ 7 min, 0.74 mg, 9.7 × 10^–4^% w/w). Subfraction 5 (*t*_R_ 24 min, 14 mg) holding epoxycoraxeniolide A (**3**) underwent repurification using T3 column 4.6 × 250 mm^2^ 5 μm (Xselect, Waters) with isocratic condition at 61%, at 1 mL/min (*t*_R_ 12 min, 0.83 mg, 1.2 × 10^–4^% w/w). Subfraction 9 (*t*_R_ 33 min, 30 mg) underwent repurification using T3 column 4.6 × 250 mm^2^ 5 μm (Xselect, Waters) with isocratic condition at 82%, at 1 mL/min, to yield known xeniolides acalycinxeniolide F (**4**) (*t*_R_ 8 min, 0.26 mg, 3.4 × 10^–5^% w/w), coraxeniolide C (**5**) (*t*_R_ 22 min, 0.73 mg, 9.6 × 10^–4^ w/w), acalycigorgin E (**6**) (*t*_R_ 23 min, 0.34 mg, 4.5 × 10^–5^% w/w), and coraxeniolide A (**7**) (t_R_ 25 min, 0.42 mg, 5.8 × 10^–5^% w/w). Subfraction 2 (t_R_ 20 min, 36.3 mg) underwent repurification using C_18_ column 4.6 × 250 mm^2^ 5 μm (Xselect, Waters) with isocratic condition at 54%, at 1 mL/min, to yield known xeniolide 9-deoxyxeniolide A (**8**) (*t*_R_ 13 min, 0.35 mg, 4.7 × 10^–5^% w/w).

#### Miolenol (**1**)

Colorless oil; [α]_D_^25^ + 19° (*c* 0.12, CH_3_CN); UV (CH_3_CN) λ_max_ (log ε) 200 (3.40) nm; ECD (*c* 3.5 × 10^–4^ M, CH_3_CN) λ_max_ (Δ*ε*) 218 (−1.42) nm; ^1^H NMR (600 MHz) and ^13^C NMR (150 MHz), Table 1; (+)-ESI-TOFMS analysis an ion peak at *m*/*z* 289.2536, calcd. for [M + H]^+^ 289.2526 (Δ + 3.6 ppm).

#### Epoxymiolenol (**2**)

Colorless oil; [α]_D_^25^ + 23° (*c* 0.12, CH_3_CN); UV (CH_3_CN) λ_max_ (log ε) 199 (3.28) nm; ECD (*c* 3.3 × 10^–4^ M, CH_3_CN) λ_max_ (Δ*ε*) 200 (−1.05) nm; ^1^H NMR (600 MHz) and ^13^C NMR (150 MHz), Table 1; (+)-ESI-TOFMS analysis an ion peak at *m*/*z* 305.2472, calcd. for [M + H]^+^ 305.2475 (Δ + 0.9 ppm).

#### Epoxycoraxeniolide A (**3**)

Colorless oil; [α]_D_^25^ + 50° (*c* 0.12, CH_3_CN); UV (CH_3_CN) λ_max_ (log ε) 199 (3.68) nm; ECD (*c* 3.1 × 10^–4^ M, CH_3_CN) λ_max_ (Δ*ε*) 199 (−4.93), 219 (+1.78) nm; ^1^H NMR (600 MHz) and ^13^C NMR (150 MHz), Table 1; (+)-ESI-TOFMS as C_20_H_30_O_3_*m*/*z* 319.2269, calcd. for [M + H]^+^ 319.2268 (Δ + 0.3 ppm).

### Computational Methods

Conformational analyses of miolenol (**1**), epoxymiolenol (**2**), and epoxycoraxeniolide A (**3**) were performed using a Monte Carlo Minimum method (MCMM) and the molecular mechanics OPLS3 force field with an energy cutoff at 5 kcal/mol in Schrodinger MacroModel.^31^ The conformational analysis was constrained by using key NOESY correlations and *J* coupling values to limit the number of conformers to only those that matched the experimental data. These conformers were then optimized and frequency calculations were performed using DFT, at the M06-2X/def2-TZVP level in Gaussian 16.^32^ Gaussian 16 was then used to calculate the ECD spectra with 50 excited states for all conformers at two separate levels: B3LYP/6-311G(2d,p) and ωB97XD/TZVP. All DFT calculations were performed using a polarizable continuum solvation model with acetonitrile as the selected solvent.^33^ The final ECD spectra were extracted, Boltzmann-weighted based on each conformer’s electronic and thermal free energy, and corrected by alignment with the UV spectra using the freely available software SpecDis 1.7.^34^

### Bioassays

Antimalarial activity was assessed with the adaptation of the sensitivity assay using SybrGreen fluorescence as an assessment of parasite growth.^35^ Control and sample compounds were prepared in 100% dimethyl sulfoxide (DMSO) at 5 mg/mL. Assays were performed in 384-well microtiter plates; each plate contained 40 μL of parasite culture (0.5% parasitemia, 2.0% hematocrit) of either NF54 wild type (WT; MRA-1000, BEI Resources) or Dd2-resistant (chloroquine, pyrimethamine and mefloquine resistant; MRA-156, BEI Resources) and 40 nL of drug dispensed using the V&P Scientific pin tool mounted to a liquid handling robot. Control wells were run with 40 nL of 5 mg/mL of a control compound (chloroquine (CQ) or dihydroartemisinin (DHA)) or 100% DMSO and 40 μL of a cell suspension. Each compound was tested with two individual replicates per concentration, and parasite growth was compared to that of controls incubated at 37 °C under normal culture conditions (5%O_2_, 5% CO_2_, 90% N_2_). Plates were frozen and thawed before adding a final 2× concentration of SybrGreen in lysis buffer and read on the CLARIOstar fluorescence plate reader (Ex/Em 484-15/528-15) following a 45 min incubation at room temperature (RT). Single-point analysis was determined after transforming the relative fluorescence response to percent inhibition using the equation (100 × (1 – (*n* – min)/(max – min))). Compounds were determined to be active if inhibition exceeded >70%.

Each compound was tested for cytotoxicity using mammalian J774A.1 cell lines (ATCC TIB-67) in complete media; RPMI medium with phenol red containing l-glutamine and then supplemented with 10% fetal bovine serum (CM). Cells were seeded at 5 × 10^5^ cells/mL and plated in a 96-well format with 100 μL/well fresh media on day 0 and were incubated overnight at 37 °C, 5% CO_2_ for adherence. Following 24 h incubation, spent media was removed and 100 μL of fresh media with test compounds were serially diluted 1:2 from a starting concentration of 10 μg/mL was added to the cells and incubated for an additional 68 h before adding in 20 μL of CellTiter 96 Aqueous One Solution Cell Proliferation Assay reagent (Promega). The cells were incubated for an additional 4 h before reading absorbance (490 nm) on the CLARIOstar plate reader. Single-point analysis was performed after transforming the response to percent inhibition using the equation (100 × (1 – (*n* – min)/(max – min))). Compounds were determined to be active if inhibition exceeded >70%.

## Data Availability

All raw data are deposited in NP-MRD at https://depositions.np-mrd.org/request-data/83f7a2ab-3beb-40f4-aef8-04d9cc991b9a.

## Supporting Information Available

All NMR data for new and known compounds **1**–**8** are provided together with HRMS and ECD data for new compounds **1**–**3** (PDF)

## Footnotes

1 *Míol mór* being the Irish Gaelic for whale, inspired by the shape of the molecule.
