# Supplementary material for: Xeniaphyllane and Xeniolide Diterpenes from the Deep-Sea Soft Coral Paragorgia arborea
Source: ACS Omega. 2024 Sep 25;9(40):41914–22. doi: 10.1021/acsomega.4c06361 (PMC11465450; doi:10.1021/acsomega.4c06361)
Supplement: Supplementary file 1 — ao4c06361_si_001.pdf [file ao4c06361_si_001.pdf]

Supplementary Information for

Xeniaphyllane and xeniolide diterpenes from the deep-sea soft coral *Paragorgia arborea*

Sam Afoullouss,[a,b,c] Ryan M. Young,[a,b] Laurence K. Jennings,[a] Jason Doyle,[a,1] Karen Croke,[b] Debora Livorsi,[d] John H. Adams,[d] Mark P. Johnson,[b] Olivier P. Thomas,[a,] A. Louise Allcock,[b,*]*

[a] *School of Biological and Chemical Sciences, Ryan Institute, University of Galway, University Road, H91TK33 Galway, Ireland*

[b] *School of Natural Sciences, Ryan Institute, University of Galway, University Road, H91TK33 Galway, Ireland;*

[c] *Department of Chemistry, University of South Florida, 4202 E. Fowler Avenue, CHE 205, Tampa, FL 33620, USA;*

[d] *Center for Global Health & Inter-disciplinary Research, College of Public Health, University of South Florida, 3720 Spectrum Blvd, STE 404, Tampa, FL 33612, USA*

[1] *Current address: CrannMed, Block 11, Galway Technology Park, Parkmore, Galway, Ireland, H91 VE0H*

Figure S1: In-situ photograph of Paragorgia arborea taken by the ROV Holland 1, at a depth of 1,500 m in Whittard canyon....4
Figure S2: Solvent used for reversed phase C_{18} vacuum liquid chromatography fractionation and Paragorgia arborea extract and the mass of each fraction in milligrams....5
Figure S3: ^{1}H NMR in $CDCl_3$ of subfraction 2 after purification with acidified solvent (top) and non-acidified solvents (bottom)....5
Figure S4: HRESIMS spectrum of miolenol (**1**)....5
Figure S5: ^{1}H NMR spectrum of miolenol (**1**) in $CDCl_3$ (600 MHz)....6
Figure S6: ^{13}C NMR spectrum of miolenol (**1**) in $CDCl_3$ (150 MHz)....7
Figure S7: gCOSY spectrum of miolenol (**1**) in $CDCl_3$ (600 MHz)....7
Figure S8: gHSQCAD spectrum of miolenol (**1**) in $CDCl_3$ (600 MHz)....8
Figure S9: gHMBCAD spectrum of miolenol (**1**) in $CDCl_3$ (600 MHz)....8
Figure S10: NOESY spectrum of miolenol (**1**) in $CDCl_3$ (600 MHz)....9
Figure S11: ^{1}H NMR spectrum of miolenol (**1**) in C_5D_5N (600 MHz)....10
Figure S12: Variable temperature ^{1}H NMR spectrum of miolenol (**1**) in C_5D_5N (600 MHz). 11
Figure S13: gCOSY spectrum of miolenol (**1**) in C_5D_5N (600 MHz)....11
Figure S14: ^{13}C NMR spectrum of miolenol (**1**) in C_5D_5N (150 MHz)....12
Figure S15:gHSQCAD spectrum of miolenol (**1**) in C_5D_5N (600 MHz)....12
Figure S16: gHMBCAD spectrum of miolenol (**1**) in C_5D_5N (600 MHz)....13
Figure S17: NOESY spectrum of miolenol (**1**) in C_5D_5N (600 MHz)....13
Figure S18:HRESIMS spectrum of epoxymiolenol (**2**)....15
Figure S19: ^{1}H NMR spectrum of epoxymiolenol (**2**) in $CDCl_3$ (600 MHz)....15
Figure S20: ^{13}C NMR spectrum of epoxymiolenol (**2**) in $CDCl_3$ (150 MHz)....16
Figure S21: gCOSY NMR spectrum of epoxymiolenol (**2**) in $CDCl_3$ (600 MHz)....16
Figure S22: gHSQCAD NMR spectrum of epoxymiolenol (**2**) in $CDCl_3$ (600 MHz)....17
Figure S23: gHMBCAD NMR spectrum of epoxymiolenol (**2**) in $CDCl_3$ (600 MHz)....17
Figure S24: NOESY NMR spectrum of epoxymiolenol (**2**) in $CDCl_3$ (600 MHz)....18
Figure S25: HRESIMS spectrum of epoxycoraxeniolide A (**3**)....19
Figure S26: ^{1}H NMR spectrum of epoxycoraxeniolide A (**3**) in $CDCl_3$ (600 MHz)....19
Figure S27: ^{13}C- NMR spectrum of epoxycoraxeniolide A (**3**) in $CDCl_3$ (150 MHz)....20
Figure S28: gCOSY NMR spectrum of epoxycoraxeniolide A (**3**) in $CDCl_3$ (600 MHz)....20
Figure S29: gHSQCAD NMR spectrum of epoxycoraxeniolide A (**3**) in $CDCl_3$ (600 MHz). 21
Figure S30: gHMBCAD NMR spectrum of epoxycoraxeniolide A (**3**) in $CDCl_3$ (600 MHz). 21
Figure S31: NOESY NMR spectrum of epoxycoraxeniolide A (**3**) in $CDCl_3$ (600 MHz)....22
Figure S32: HRESIMS spectrum of coraxeniolide A (**4**)....23
Figure S33: ^{1}H NMR spectrum coraxeniolide A (**4**) of in $CDCl_3$ (600 MHz)....23
Figure S34: gHSQCAD spectrum coraxeniolide A (**4**) of in $CDCl_3$ (600 MHz)....24
Figure S35: HRESIMS spectrum of coraxeniolide B (**5**)....25
Figure S36: ^{1}H NMR spectrum coraxeniolide B (**5**) of in $CDCl_3$ (600 MHz)....25
Figure S37: gHSQCAD NMR spectrum coraxeniolide B (**5**) of in $CDCl_3$ (600 MHz)....26
Figure S38: HRESIMS spectrum of acalycixeniolide F (**6**)....27
Figure S39: ^{1}H NMR spectrum of acalycixeniolide F (**6**) in $CDCl_3$ (600 MHz)....27
Figure S40: gCOSY NMR spectrum of acalycixeniolide F (**6**) in $CDCl_3$ (600 MHz)....28
Figure S41: gHSQCAD NMR spectrum of acalycixeniolide F (**6**) in $CDCl_3$ (600 MHz)....28

Figure S42: (+)HRESIMS spectrum acalycigorgin E (**7**). 29
Figure S43:^{1}H NMR spectrum acalycigorgin E (**7**) of in $CDCl_3$ (600 MHz). 29
Figure S44: gHSQCAD NMR spectrum acalycigorgin E (**7**) of in $CDCl_3$ (600 MHz). 30
Figure S45:HRESIMS spectrum of 9-deoxyxeniolide A (**8**). 31
Figure S46: ^{1}H NMR spectrum 9-deoxyxeniolide A (**8**) of in $CDCl_3$ (600 MHz)..................31
Figure S47: ^{13}C NMR spectrum of 9-deoxyxeniolide A (**8**) of in $CDCl_3$ (150 MHz).............32
Figure S48: gHSQCAD NMR spectrum of 9-deoxyxeniolide A (**8**) of in $CDCl_3$ (600 MHz).32
Figure S49: Calculated and experimental ECD spectra for epoxymiolenol (**2**) measured in acetonitrile..33
Figure S50 Calculated and experimental ECD spectra for epoxycoraxenolide (**3**) measured in acetonitrile..33

Table S1: ^{1}H NMR (600 MHz) and ^{13}C NMR (150 MHz) spectroscopic data for miolenol (**1**) in deuterated pyridine…………………………………………………..……13

Table S2: Antiplasmodial activities (% inhibition) of compounds **1**-**8**, tested against NF54 and Dd2……………………………..…………………………………………………33

Table S3: Antiplasmodial activities (µM) of compounds **1**-**8**, tested against NF54 and Dd2..33

Table S4: Conformer generation constraints informed by experimental NOESY correlations and coupling constant values…………………………………………………………..34

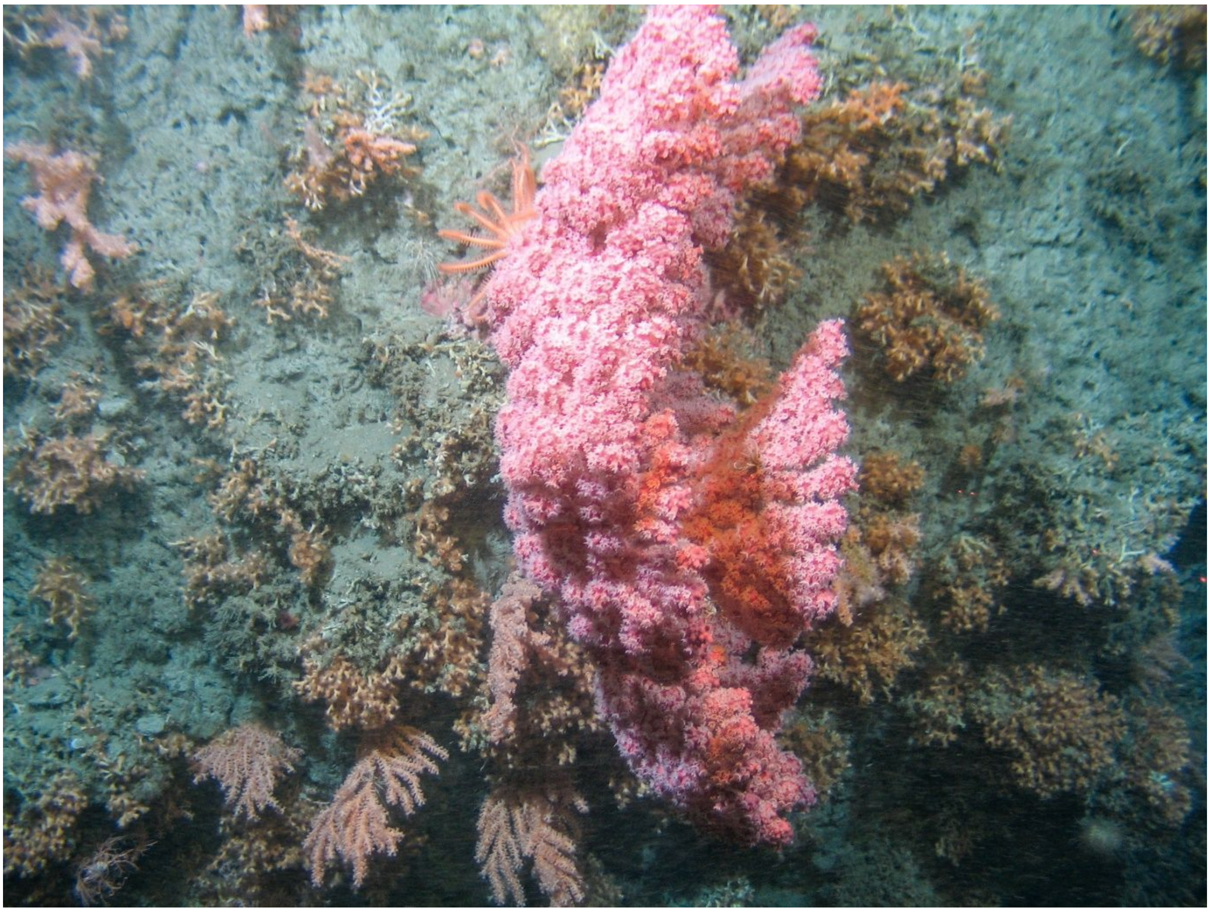

Figure S1: *In-situ* photograph of *Paragorgia arborea* taken by the ROV *Holland 1*, at a depth of 1,500 m in Whittard canyon.

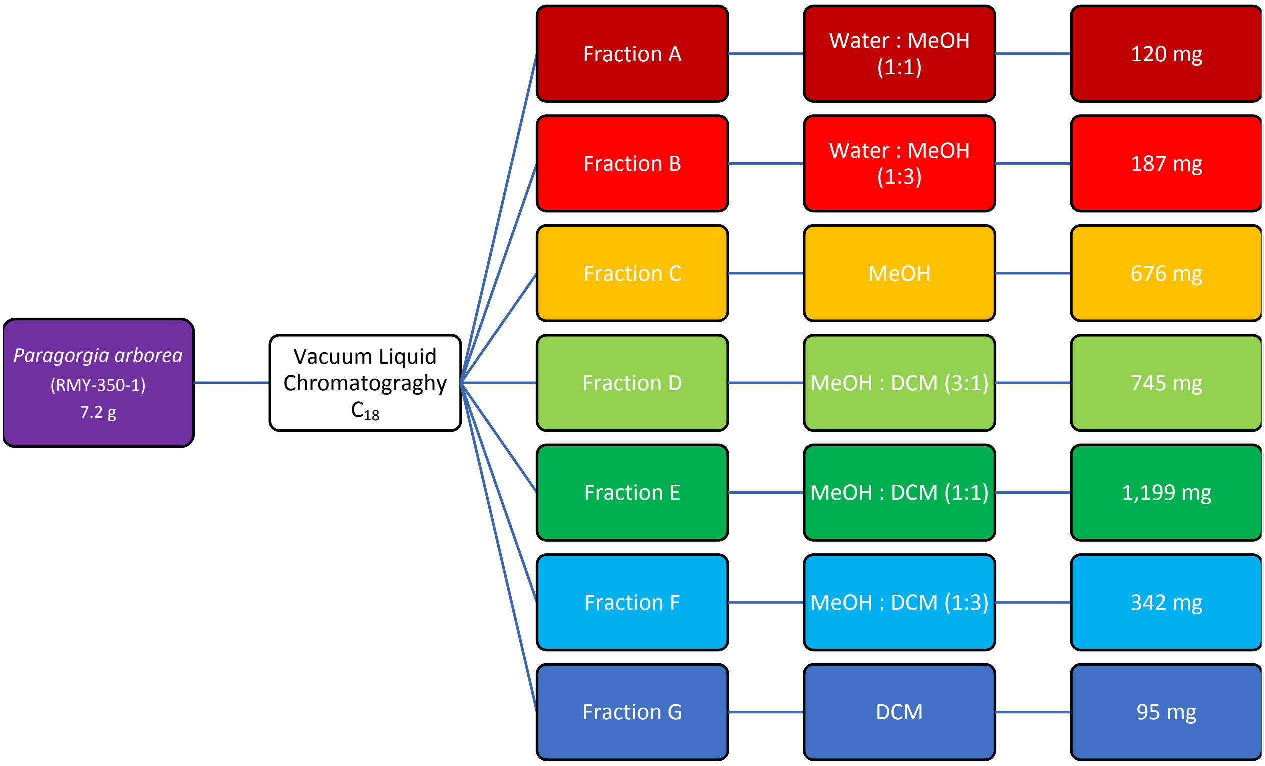

Figure S2: Solvent used for reversed phase C_{18} vacuum liquid chromatography fractionation and *Paragorgia arborea* extract and the mass of each fraction in milligrams.

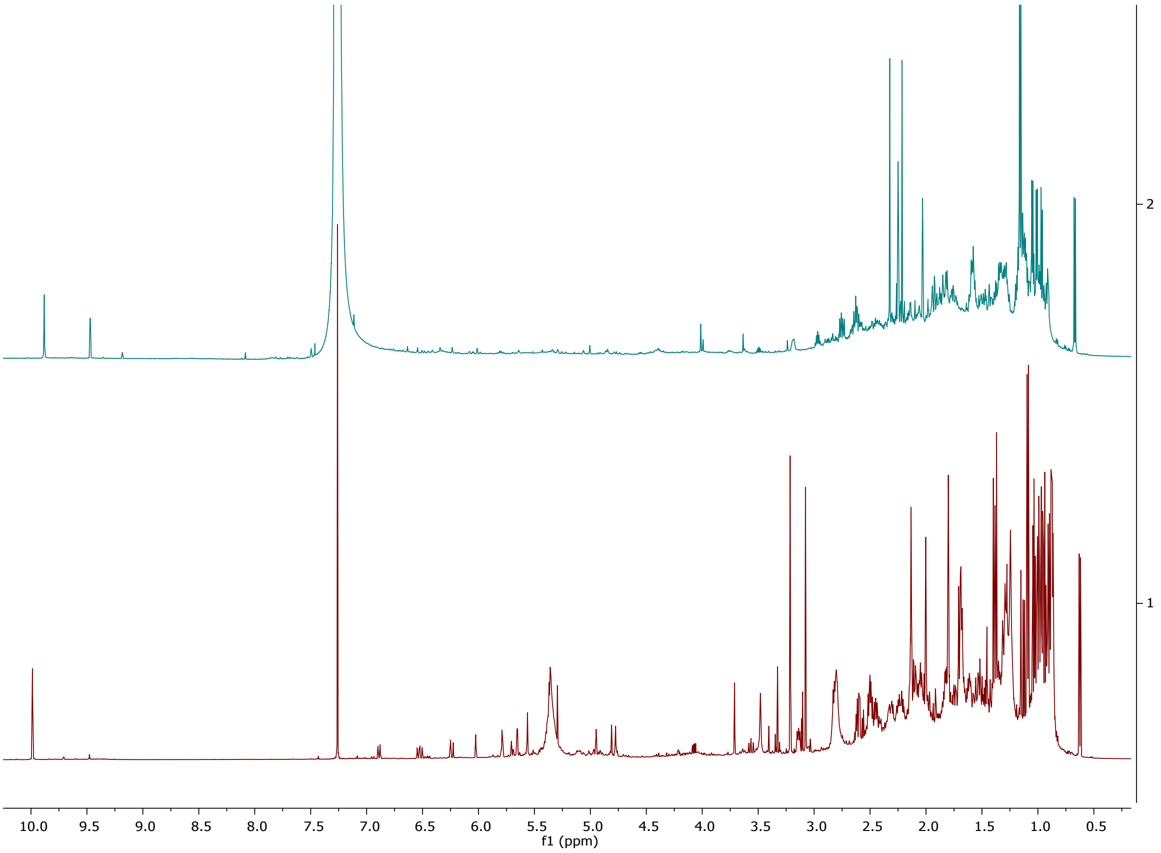

Figure S3: ^{1}H NMR in $CDCl_3$ of subfraction 2 after purification with acidified solvent (top) and non-acidified solvents (bottom).

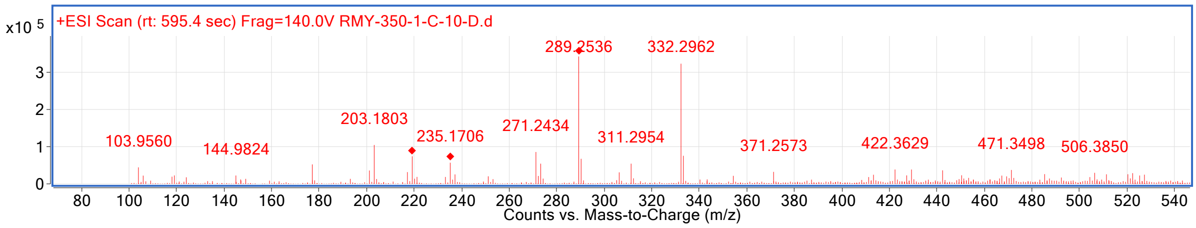

Figure S4: HRESIMS spectrum of miolenol (**1**).

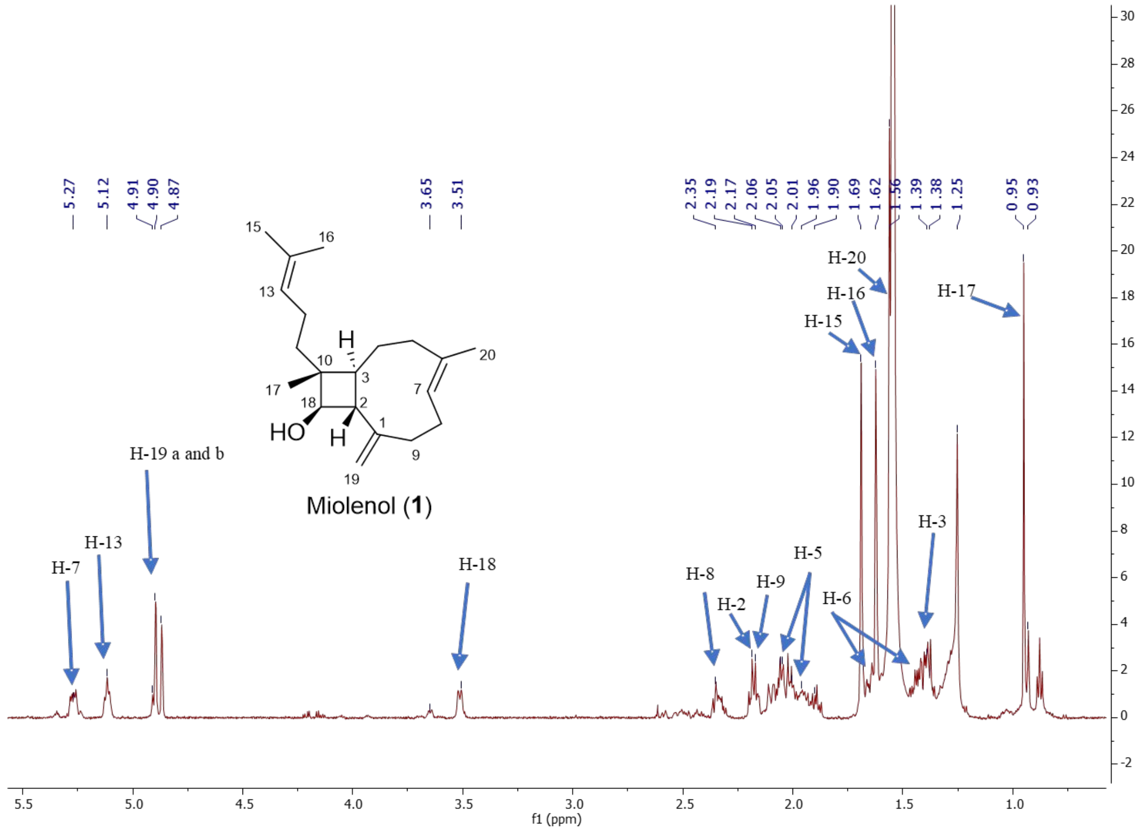

Figure S5: ^{1}H NMR spectrum of miolenol (1) in $CDCl_3$ (600 MHz).

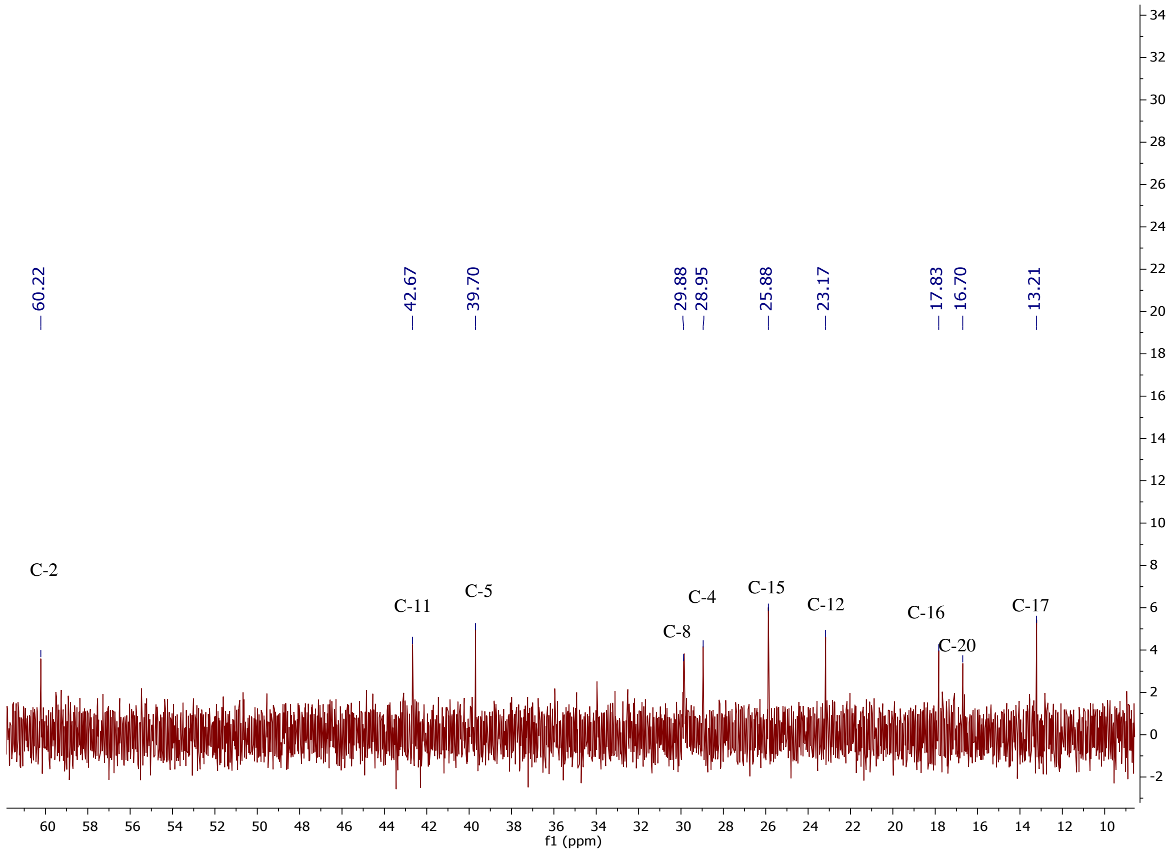

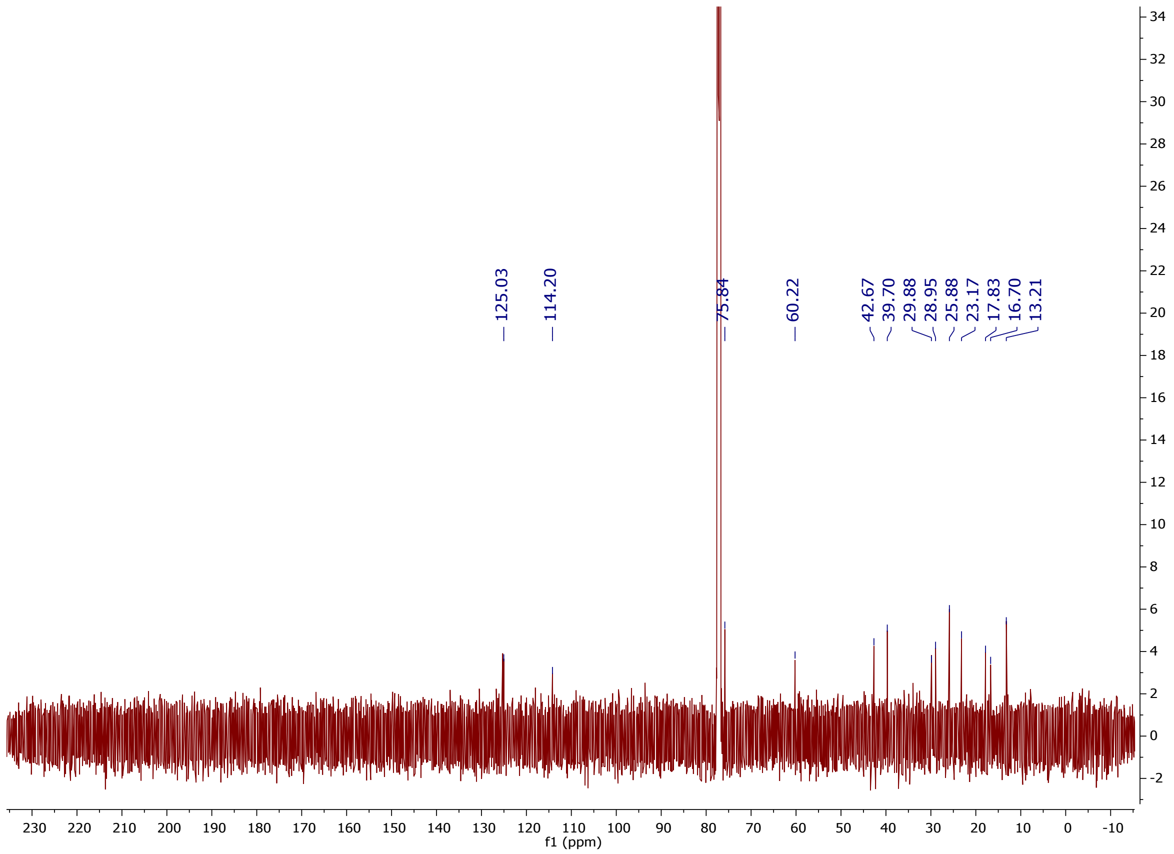

Figure S6: ^{13}C NMR spectrum of miolenol (**1**) in $CDCl_3$ (150 MHz).

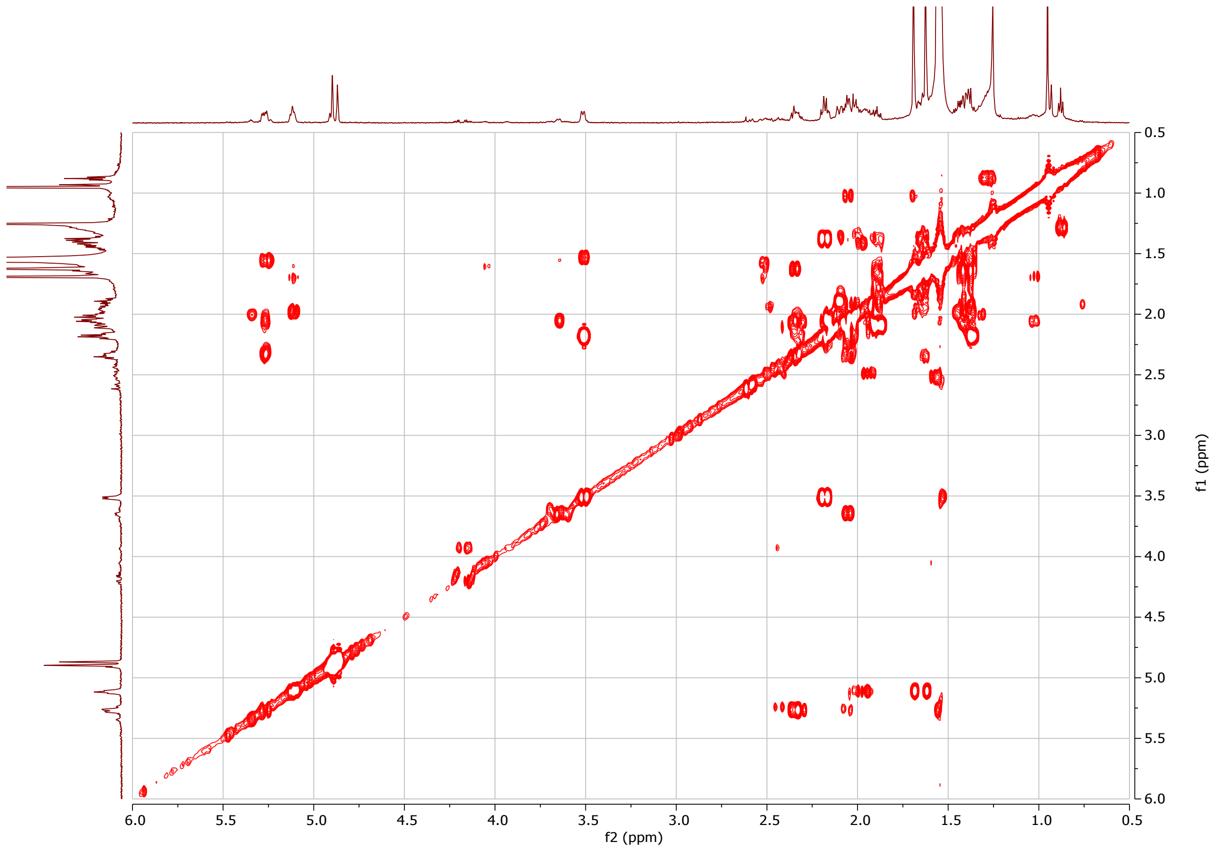

Figure S7: gCOSY spectrum of miolenol (**1**) in $CDCl_3$ (600 MHz).

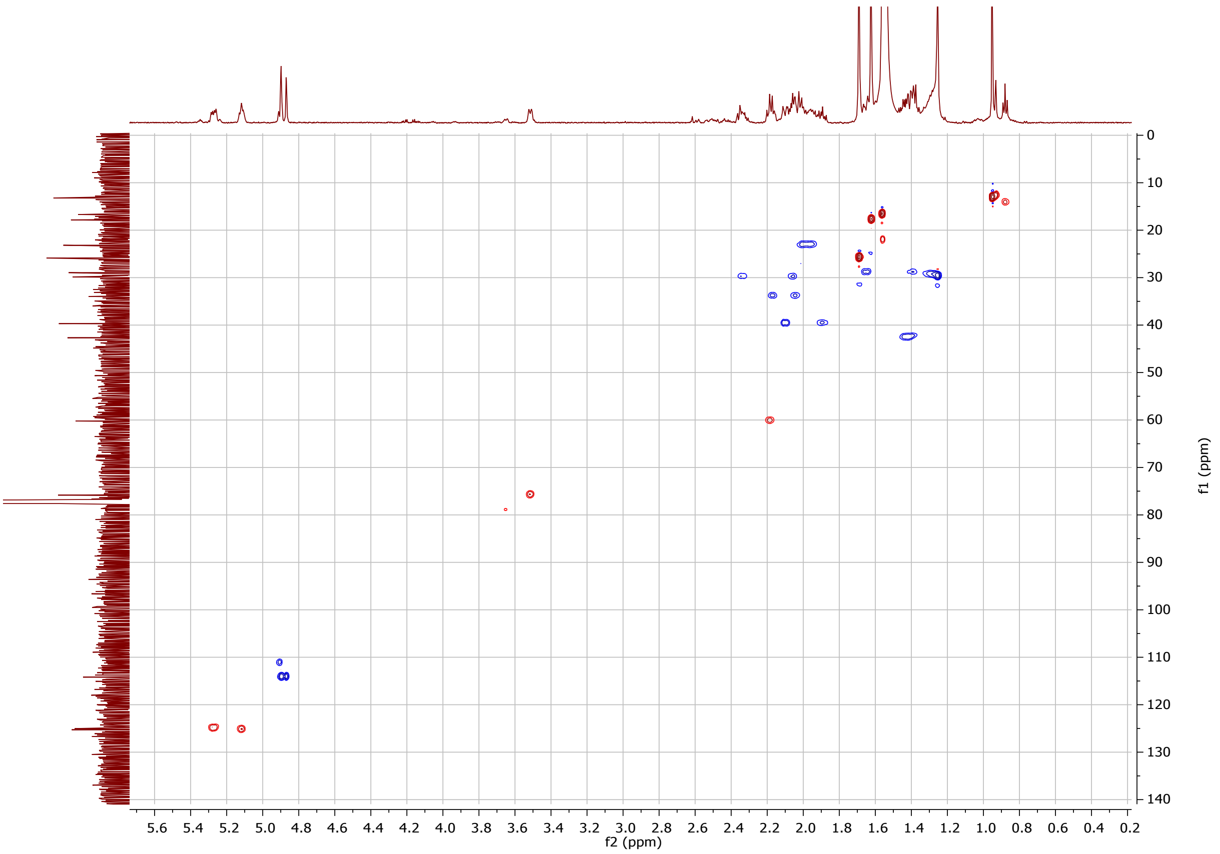

Figure S8: gHSQCAD spectrum of miolenol (**1**) in $CDCl_3$ (600 MHz).

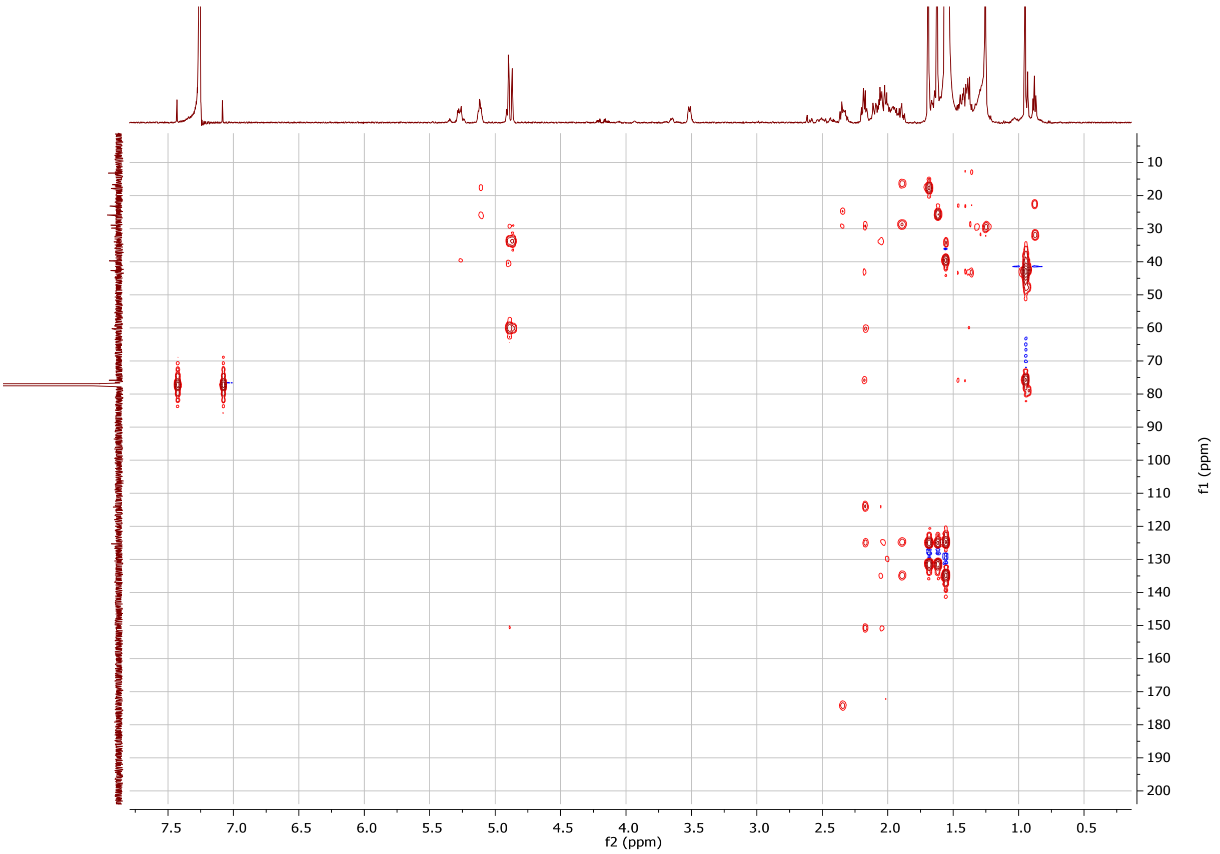

Figure S9: gHMBCAD spectrum of miolenol (**1**) in $CDCl_3$ (600 MHz).

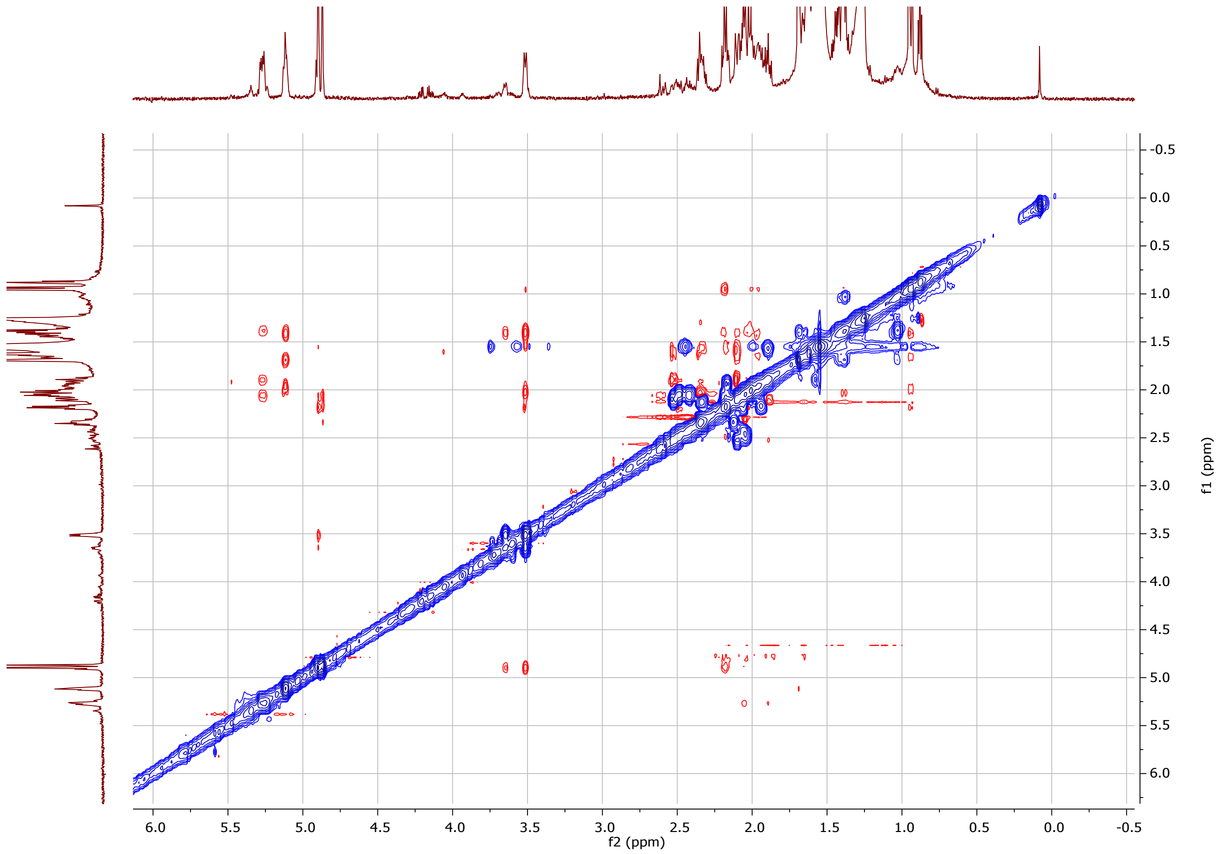

Figure S10: NOESY spectrum of miolenol (**1**) in $CDCl_3$ (600 MHz).

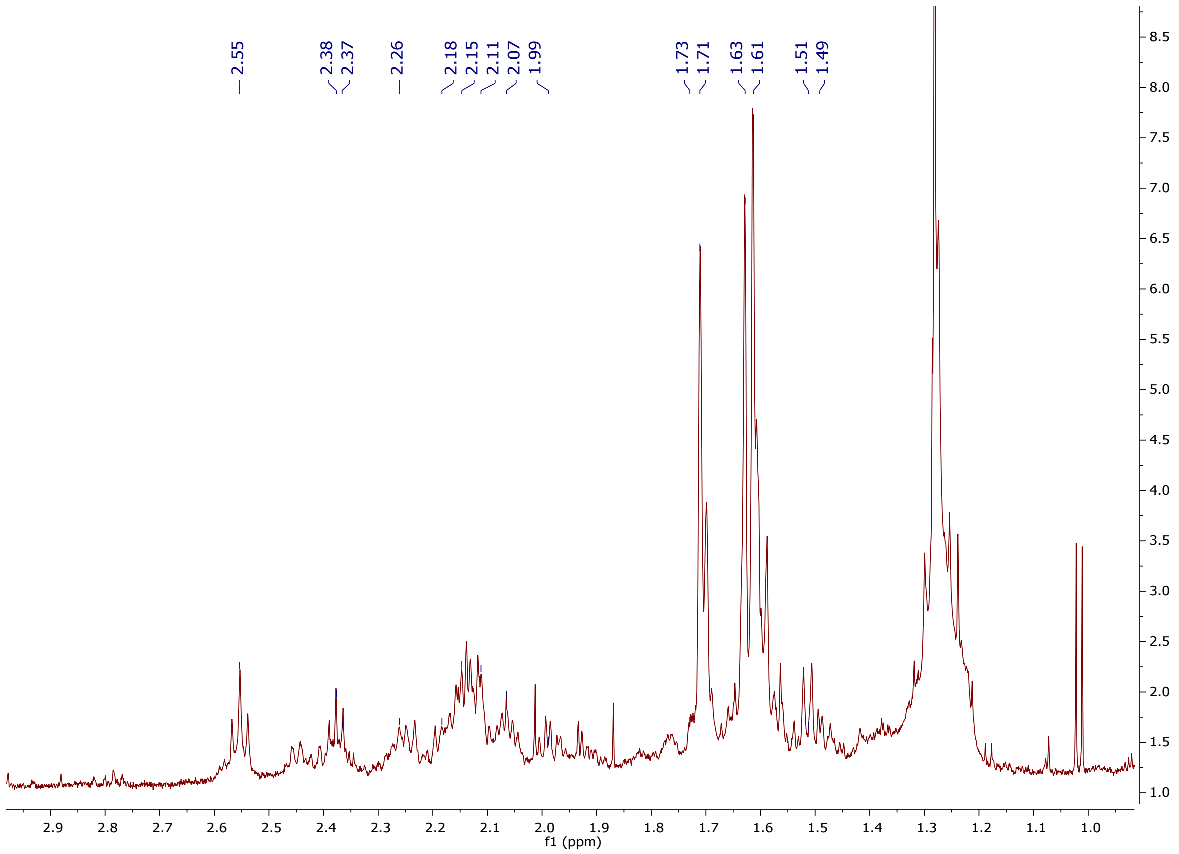

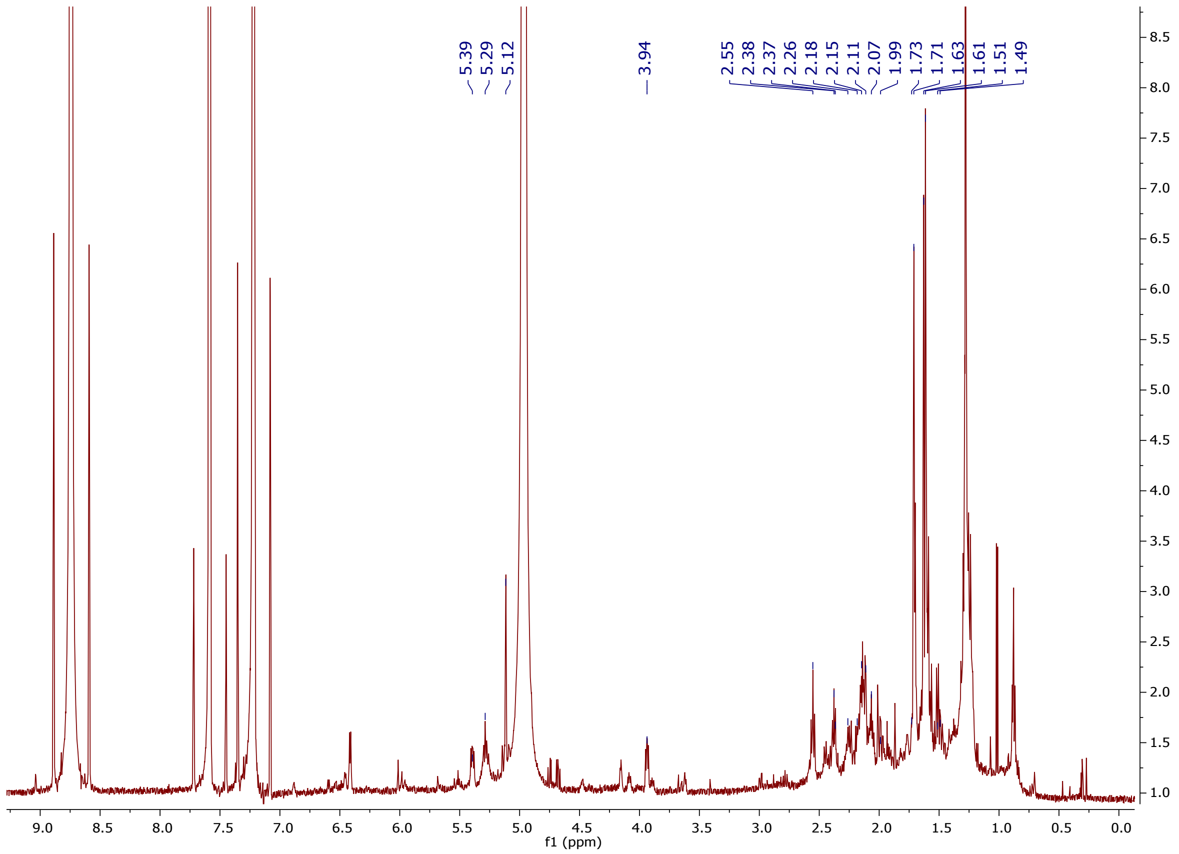

Figure S11: ^{1}H NMR spectrum of miolenol (**1**) in C_5D_5N (600 MHz).

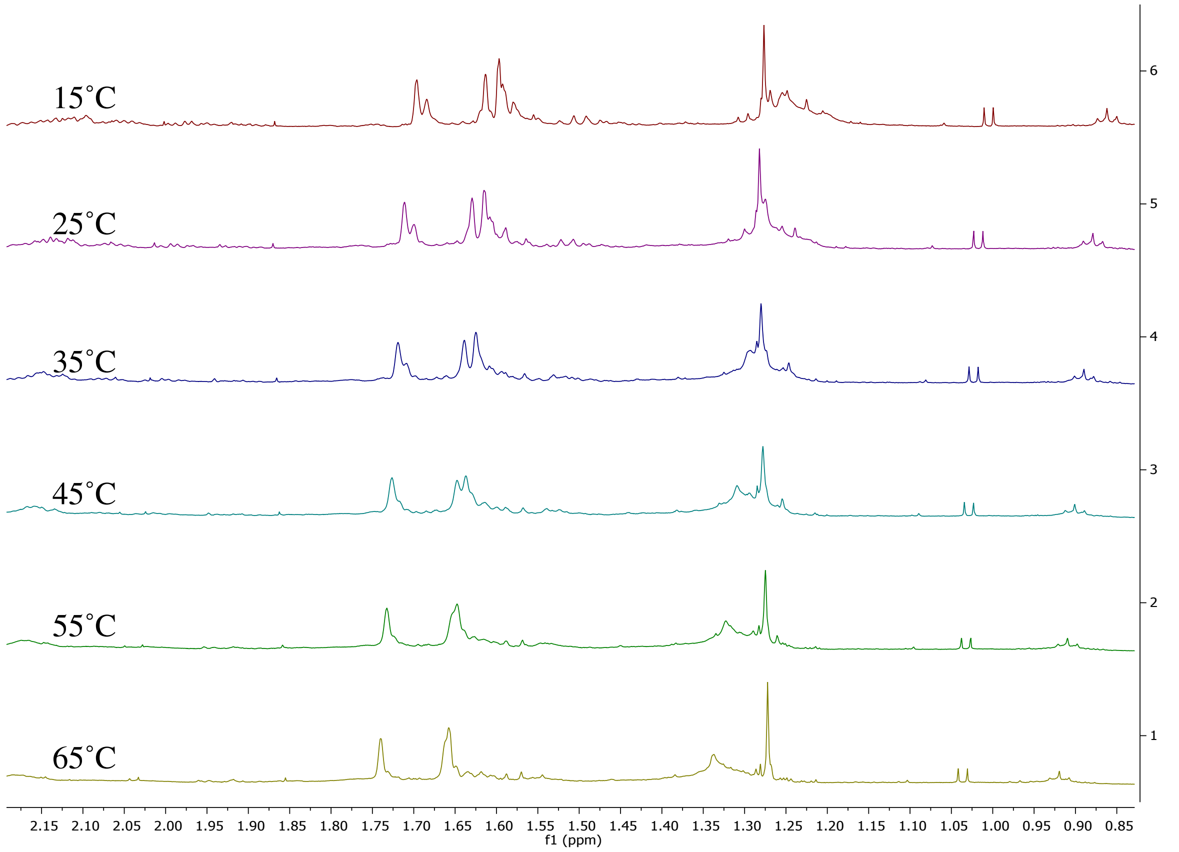

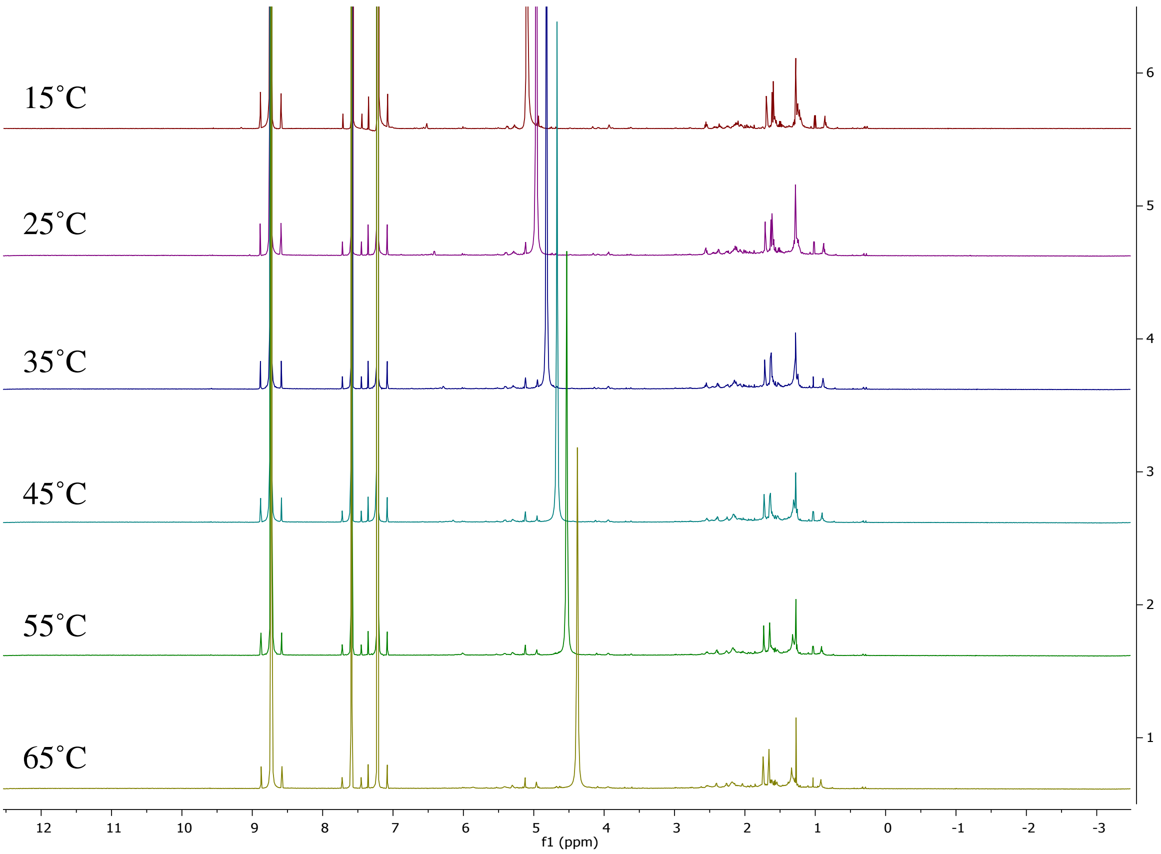

Figure S12: Variable temperature ^{1}H NMR spectrum of miolenol (**1**) in C_5D_5N (600 MHz).

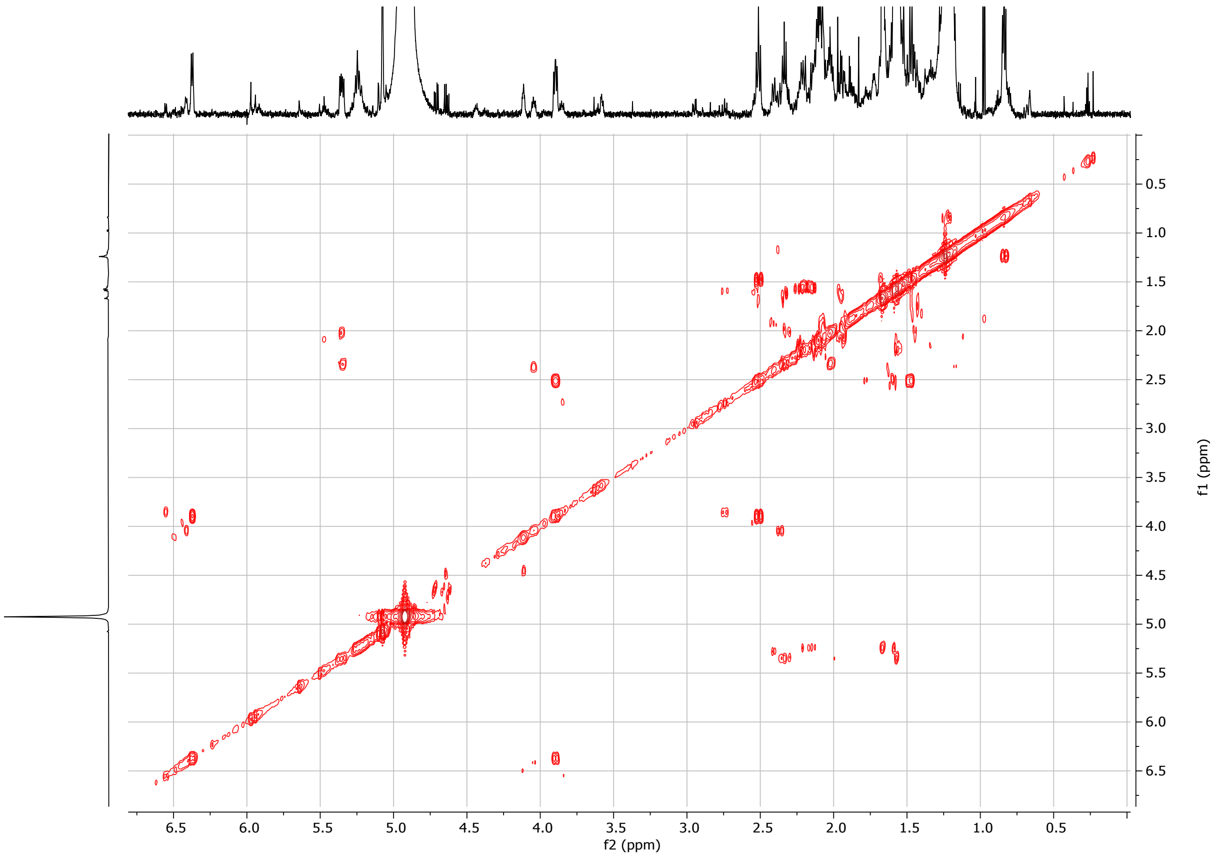

Figure S13: gCOSY spectrum of miolenol (**1**) in C_5D_5N (600 MHz).

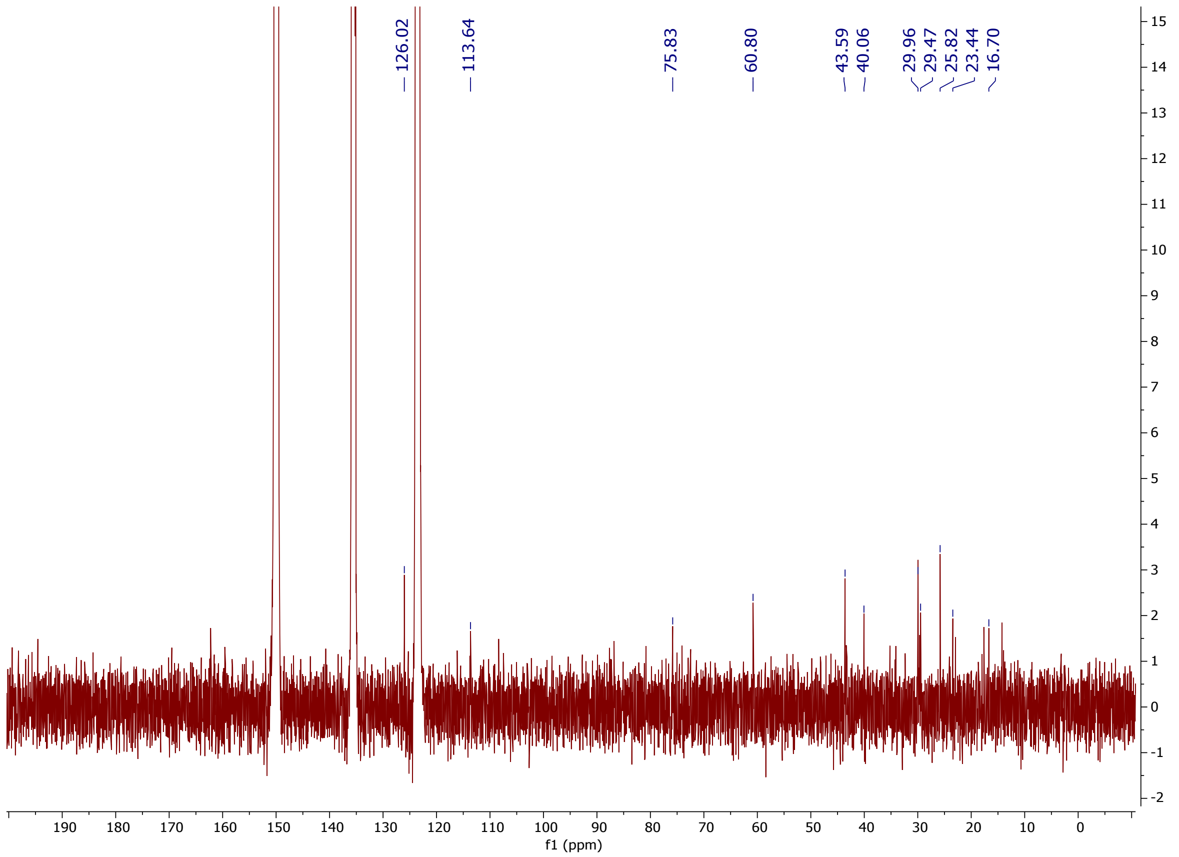

Figure S14: ^{13}C NMR spectrum of miolenol (1) in C_5D_5N (150 MHz).

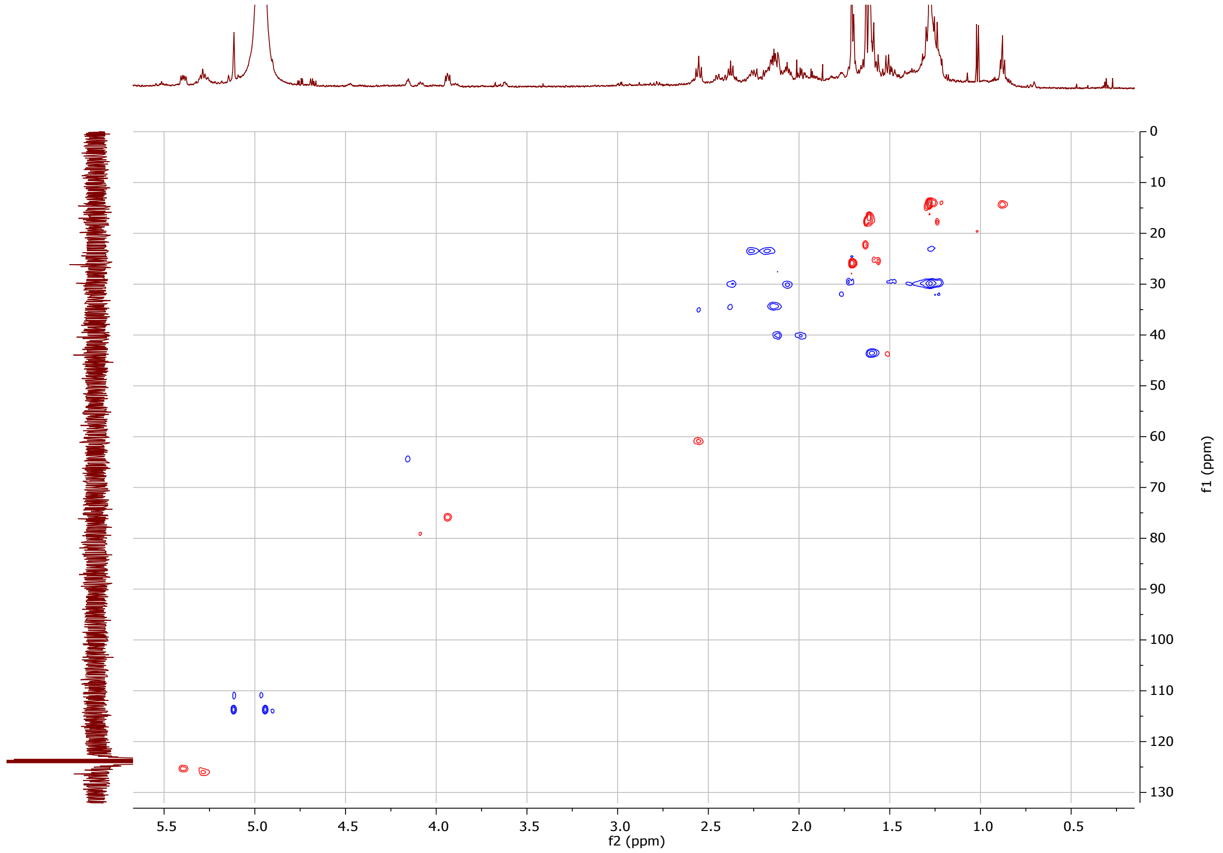

Figure S15:gHSQCAD spectrum of miolenol (1) in C_5D_5N (600 MHz).

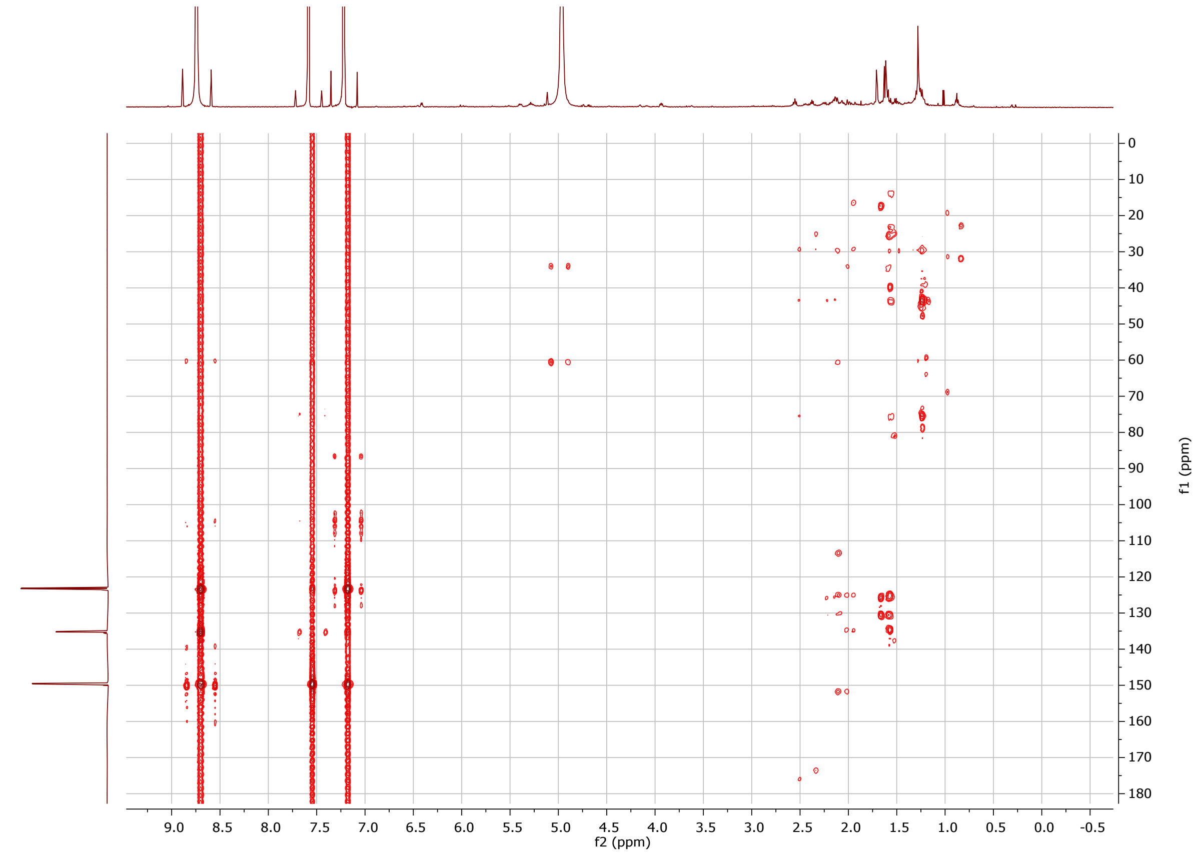

Figure S16: gHMBCAD spectrum of miolenol (**1**) in C_5D_5N (600 MHz).

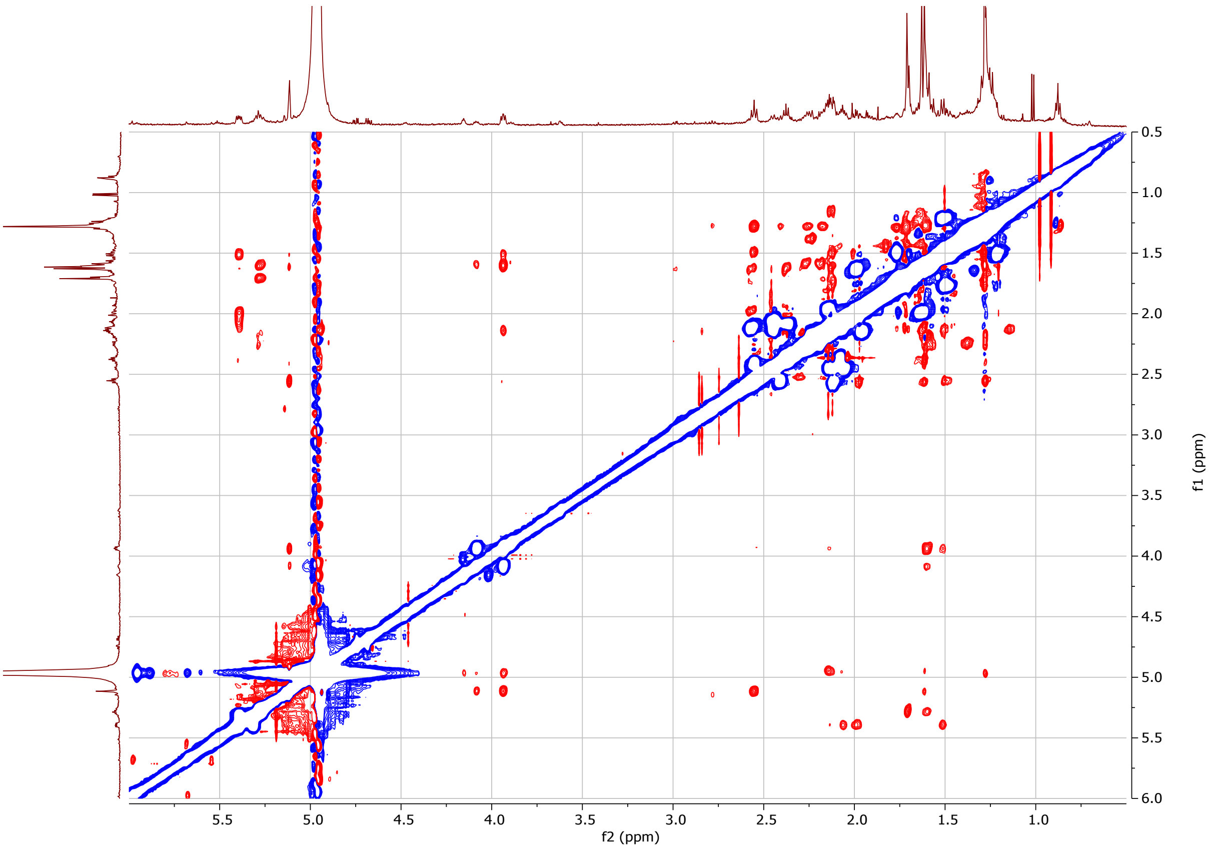

Figure S17: NOESY spectrum of miolenol (**1**) in C_5D_5N (600 MHz).

Pos	δ_C, Type	δ_H mult. (J in Hz)
1	152.5, C	-
2	60.8, CH	2.55 m
3	43.8, CH	1.51 m
4	29.5, CH_2	1.73 m, 1.49 m
5	40.1, CH_2	2.11 m, 1.99 m
6	135.3, C	-
7	125.3, CH	5.39 dd (11.0, 6.0)
8	30.1, CH_2	2.37 m, 2.07 m
9	34.4, CH_2	2.38 m, 2.15 m
10	44.1, C	-
11	43.6, CH_2	1.60 m
12	23.5, CH_2	2.26 m, 2.18 m
13	126.0, CH	5.29 t (7.5)
14	131.1, C	-
15	25.9, CH_3	1.71 s
16	22.3, CH_3	1.63 s
17	16.8, CH_3	0.95 s
18	75.9, CH	3.94 d (7.0)
19	113.7, CH_2	5.12 br s, 4.94 br s
20	16.8, CH_3	1.61 s

Table S1: ^{1}H NMR (600 MHz) and ^{13}C NMR (150 MHz) spectroscopic data for miolenol (1) in deuterated pyridine,

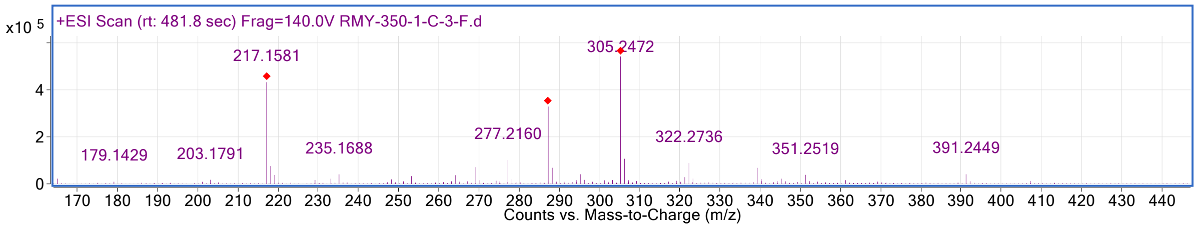

Figure S18:HRESIMS spectrum of epoxymiolenol (**2**).

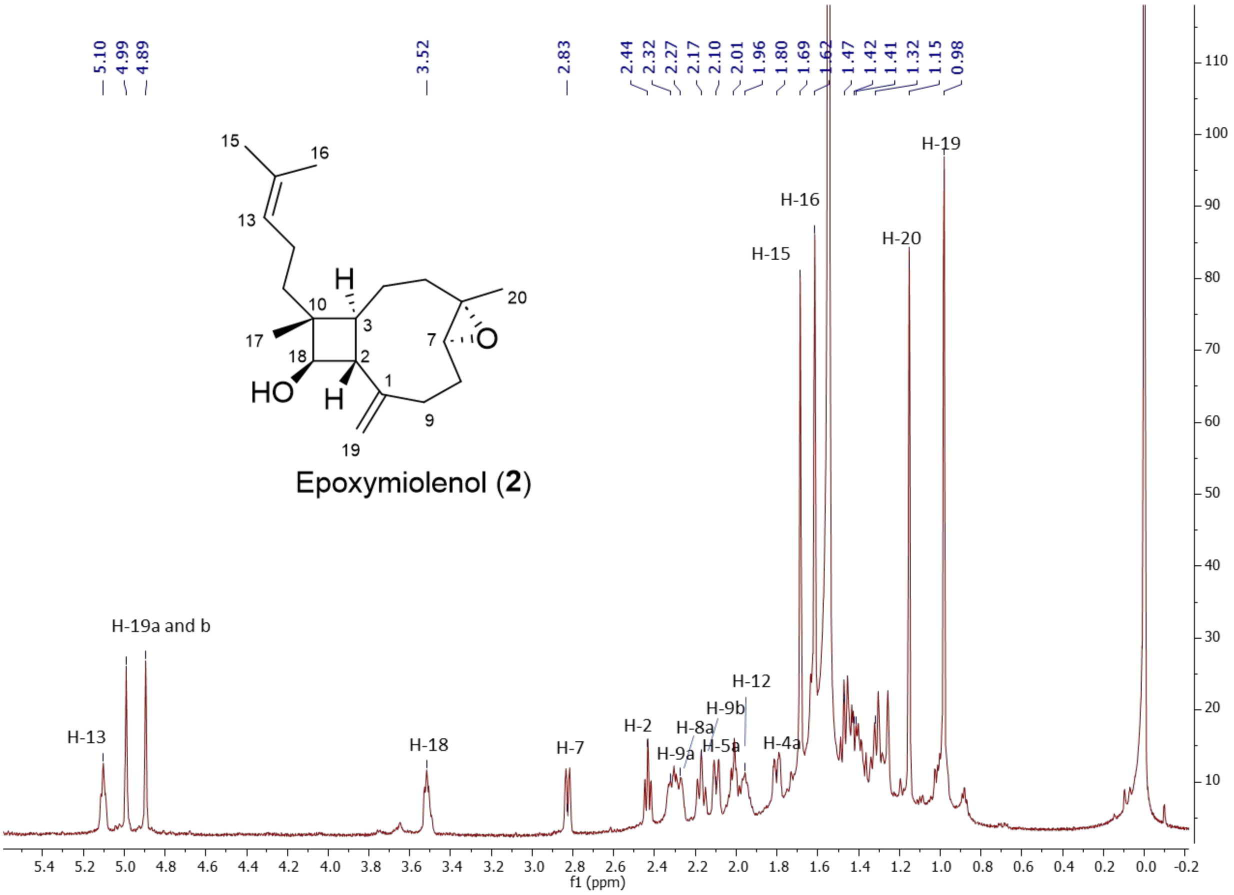

Figure S19: ^{1}H NMR spectrum of epoxymiolenol (**2**) in $CDCl_3$ (600 MHz).

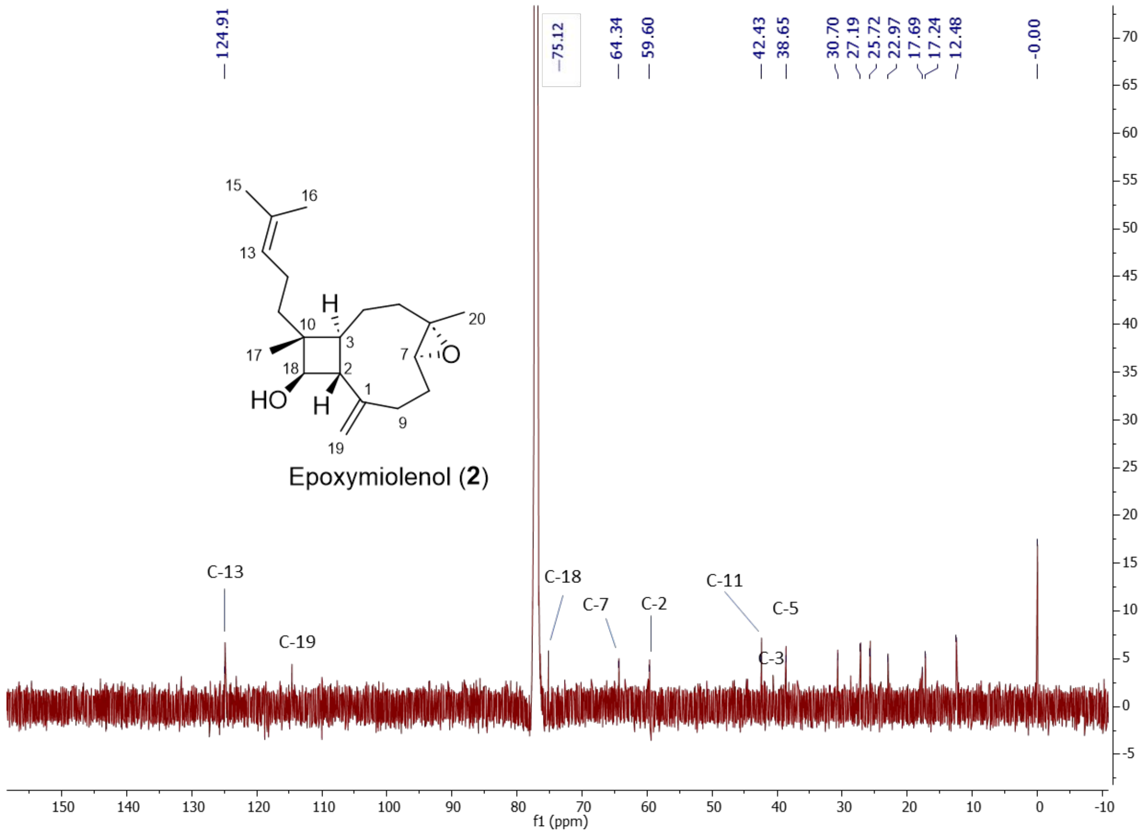

Figure S20: ^{13}C NMR spectrum of epoxymiolenol (**2**) in $CDCl_3$ (150 MHz).

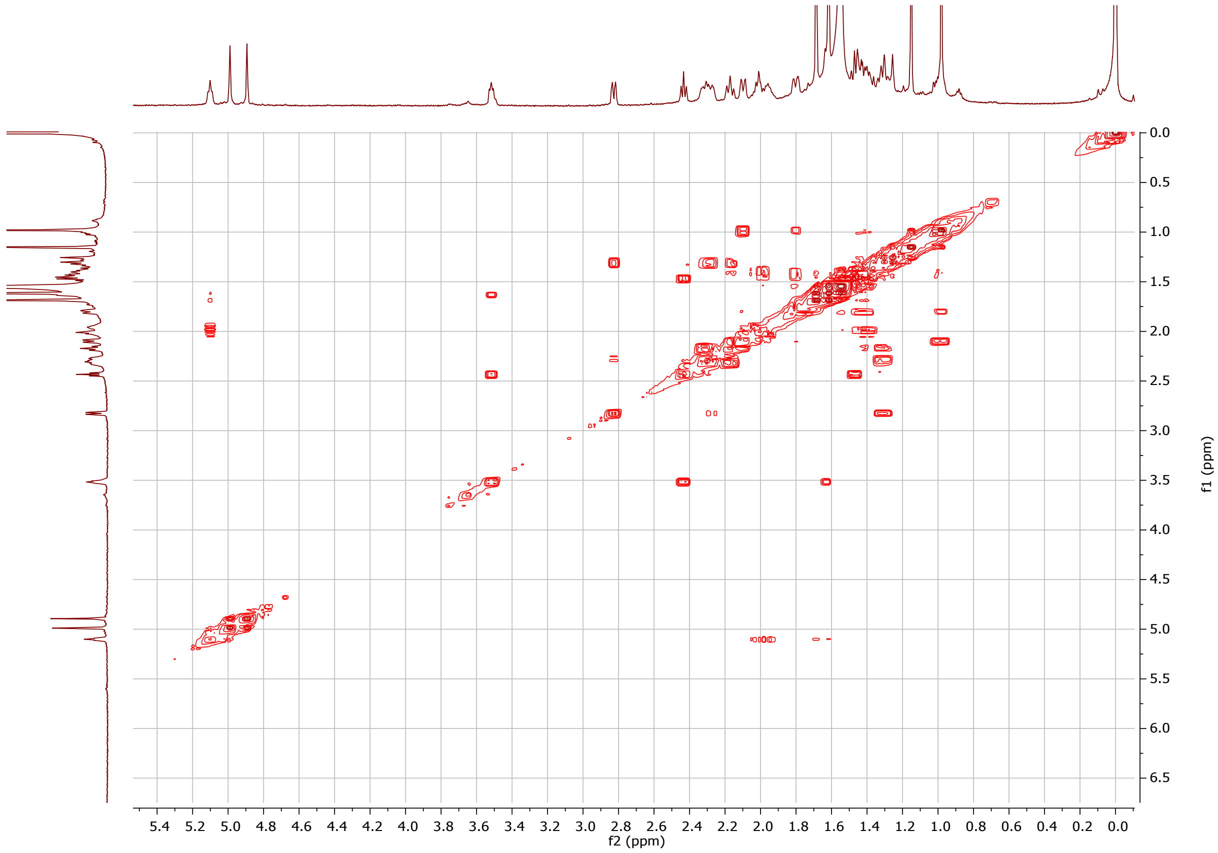

Figure S21: gCOSY NMR spectrum of epoxymiolenol (**2**) in $CDCl_3$ (600 MHz).

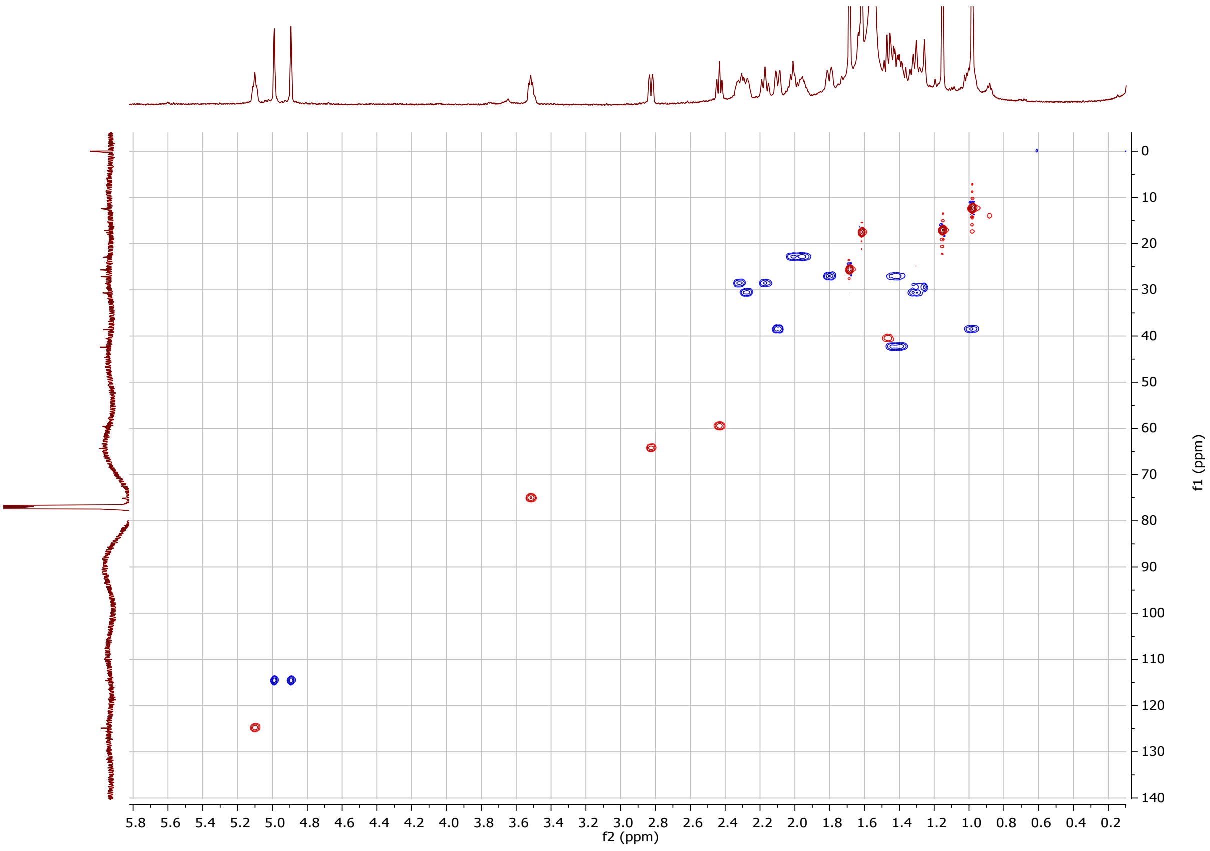

Figure S22: gHSQCAD NMR spectrum of epoxymiolenol (**2**) in $CDCl_3$ (600 MHz).

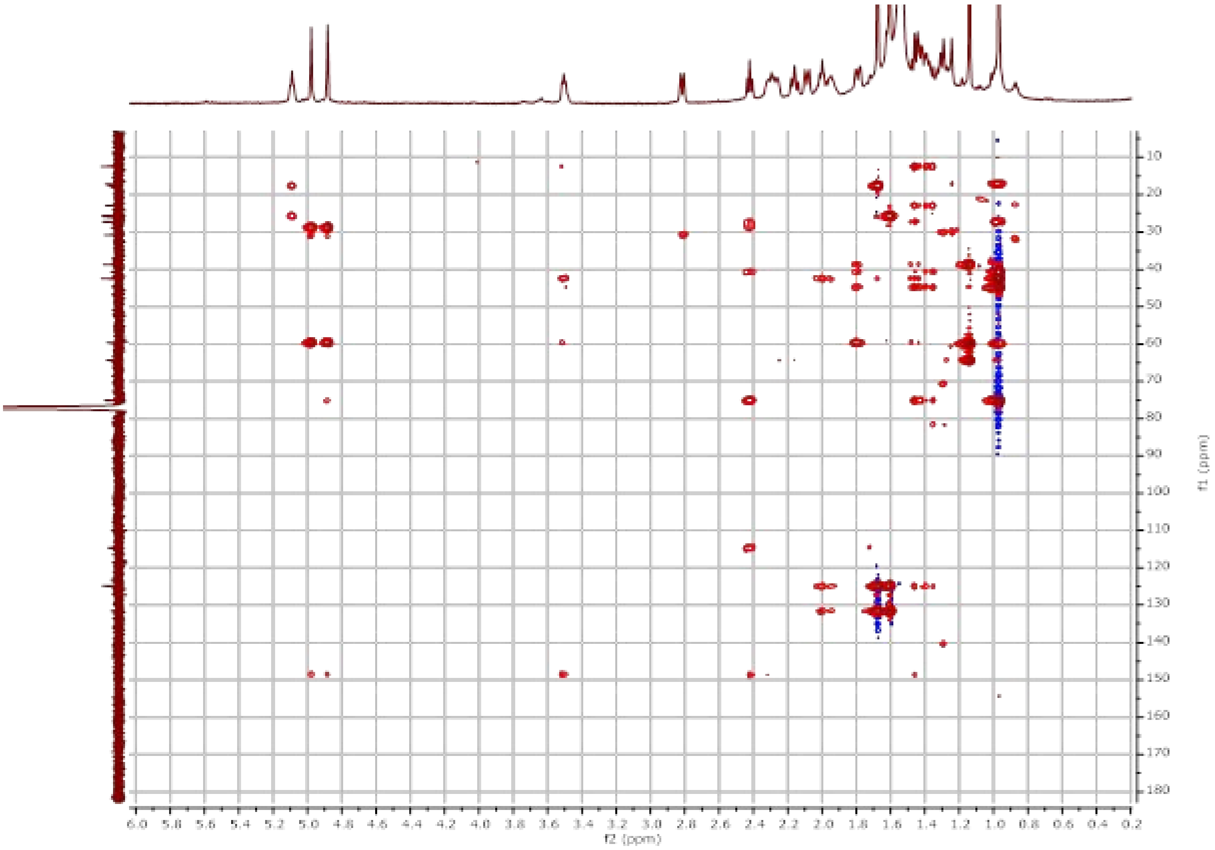

Figure S23: gHMBCAD NMR spectrum of epoxymiolenol (**2**) in $CDCl_3$ (600 MHz).

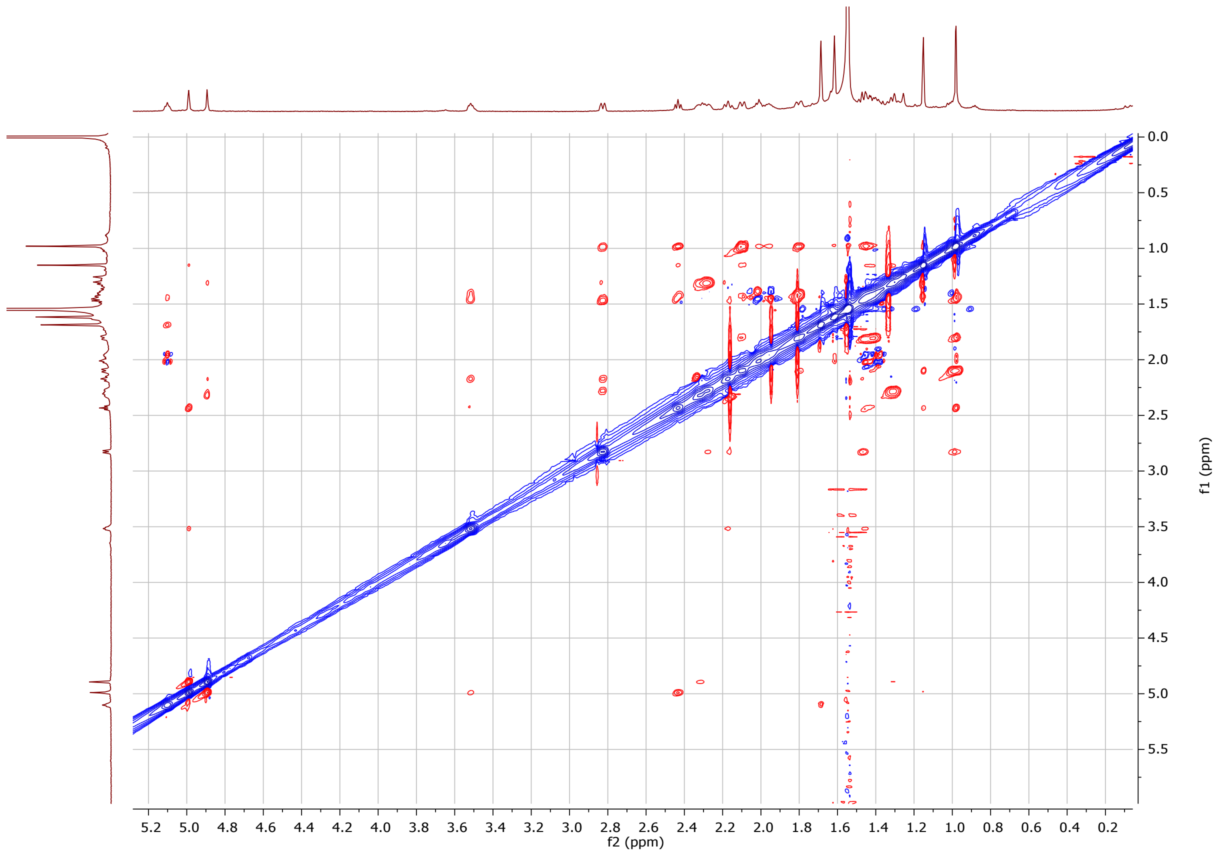

Figure S24: NOESY NMR spectrum of epoxymiolenol (**2**) in $CDCl_3$ (600 MHz).

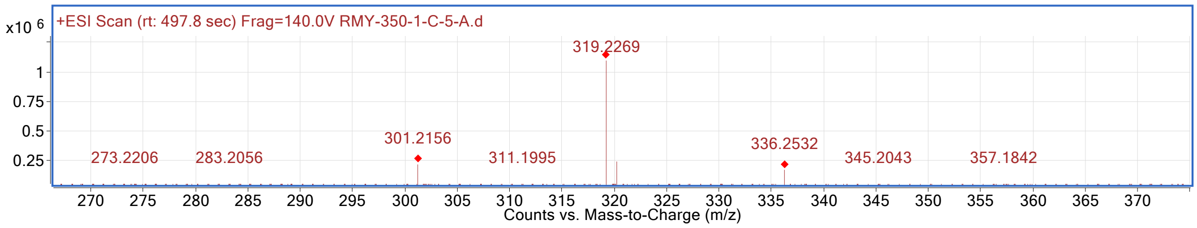

Figure S25: HRESIMS spectrum of epoxycoraxeniolide A (**3**).

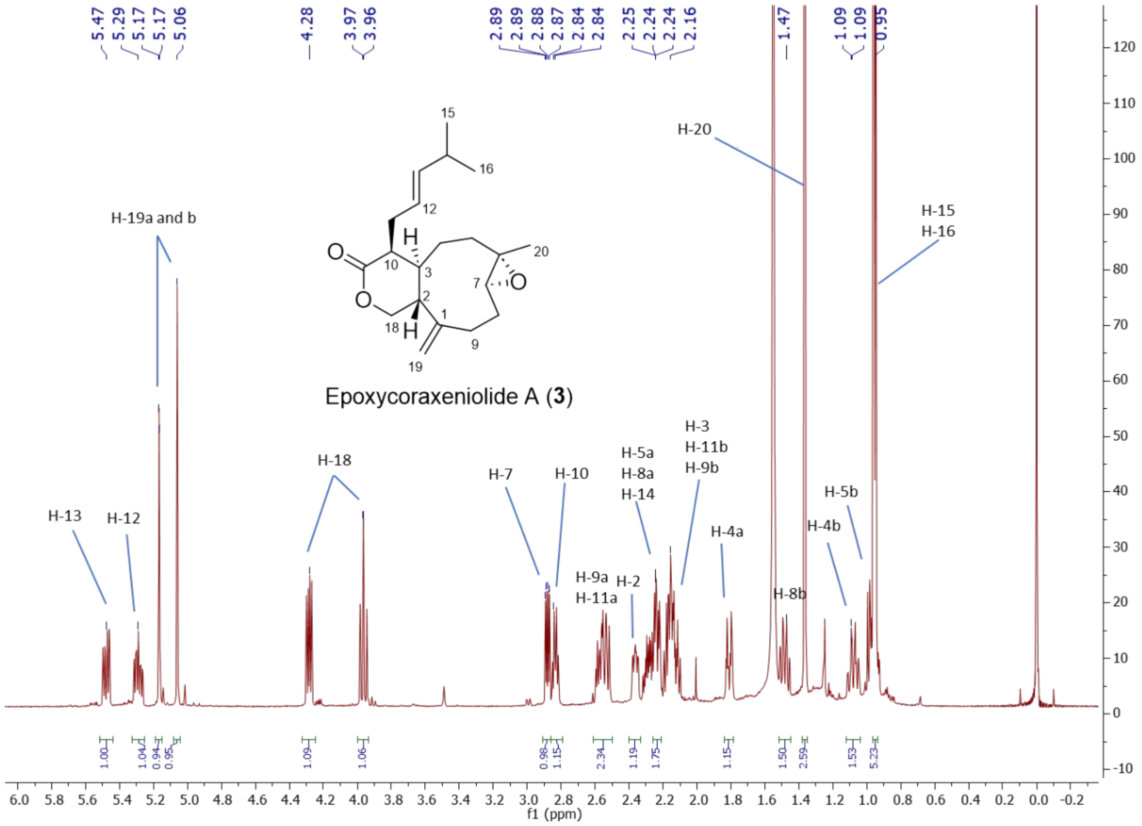

Figure S26: ^{1}H NMR spectrum of epoxycoraxeniolide A (**3**) in $CDCl_3$ (600 MHz).

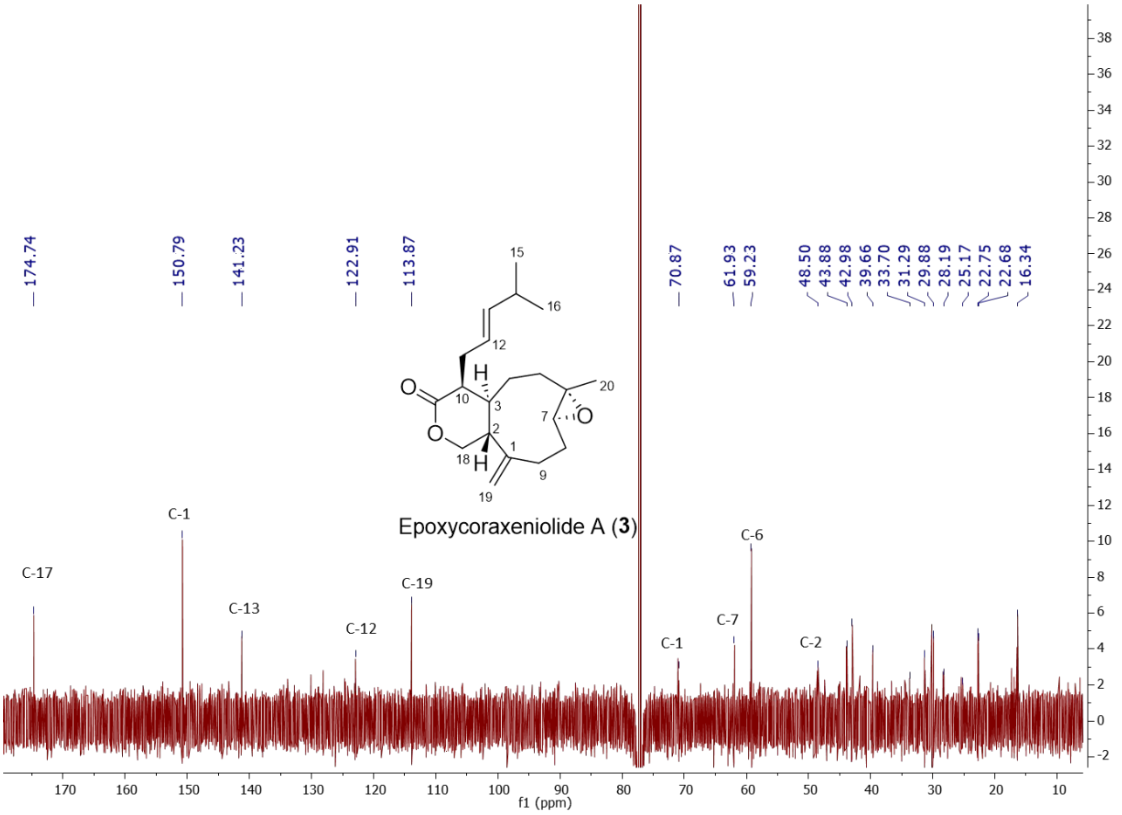

Figure S27: ^{13}C- NMR spectrum of epoxycoraxeniolide A (3) in $CDCl_3$ (150 MHz).

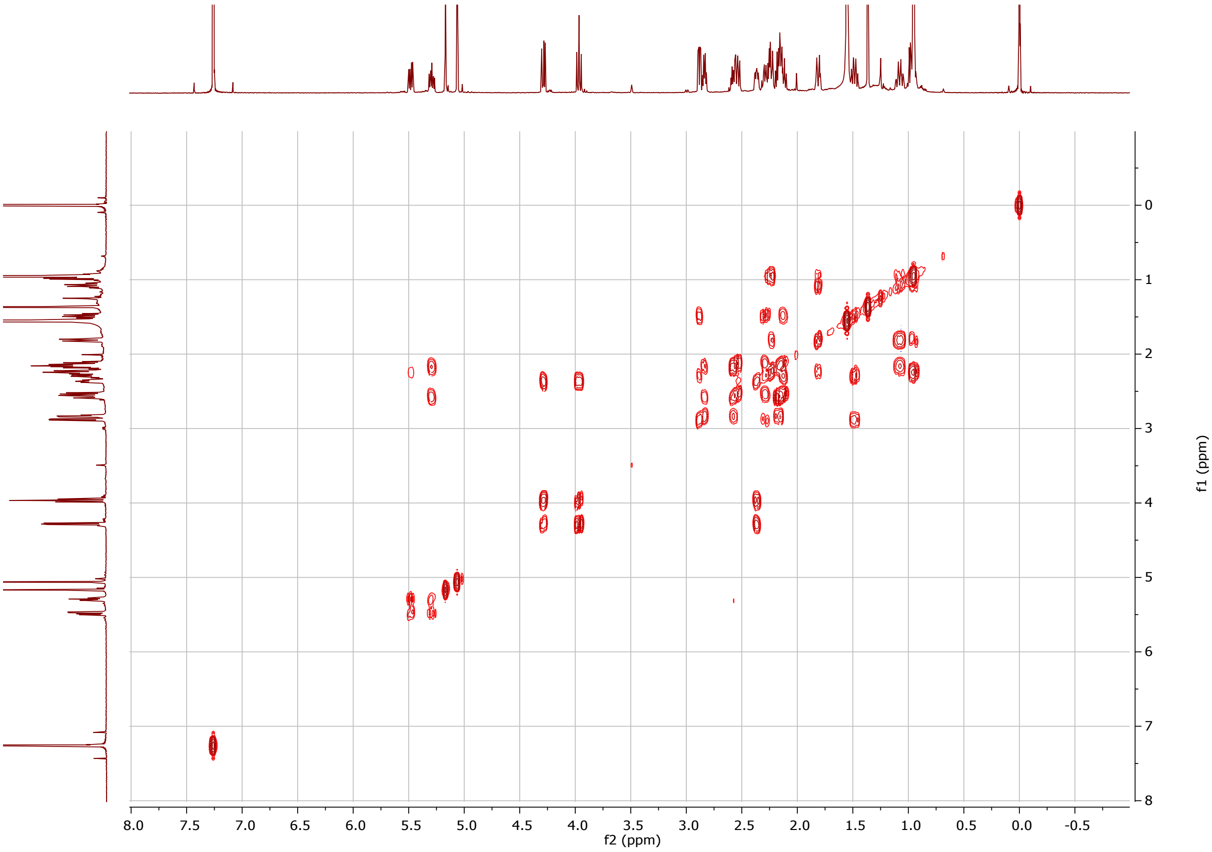

Figure S28: gCOSY NMR spectrum of epoxycoraxeniolide A (3) in $CDCl_3$ (600 MHz).

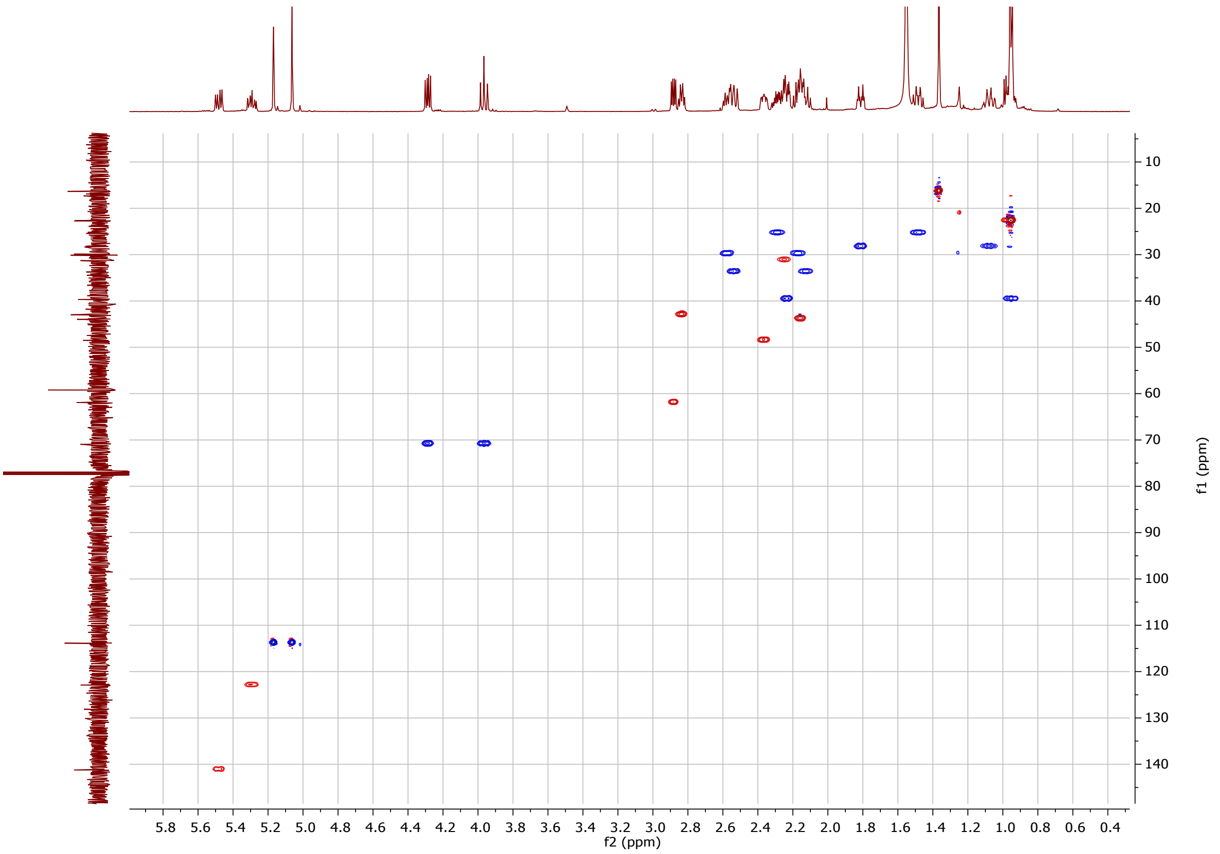

Figure S29: gHSQCAD NMR spectrum of epoxycoraxeniolide A (**3**) in $CDCl_3$ (600 MHz).

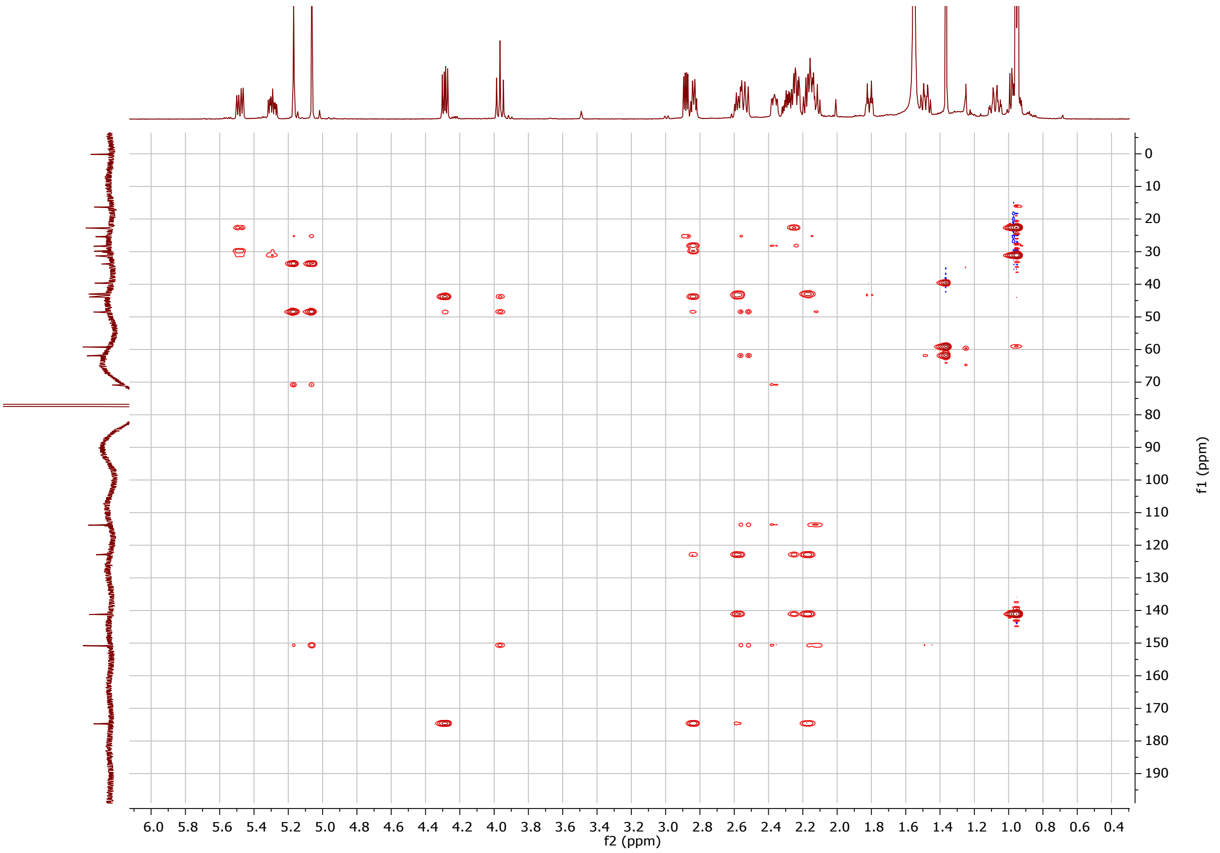

Figure S30: gHMBCAD NMR spectrum of epoxycoraxeniolide A (**3**) in $CDCl_3$ (600 MHz).

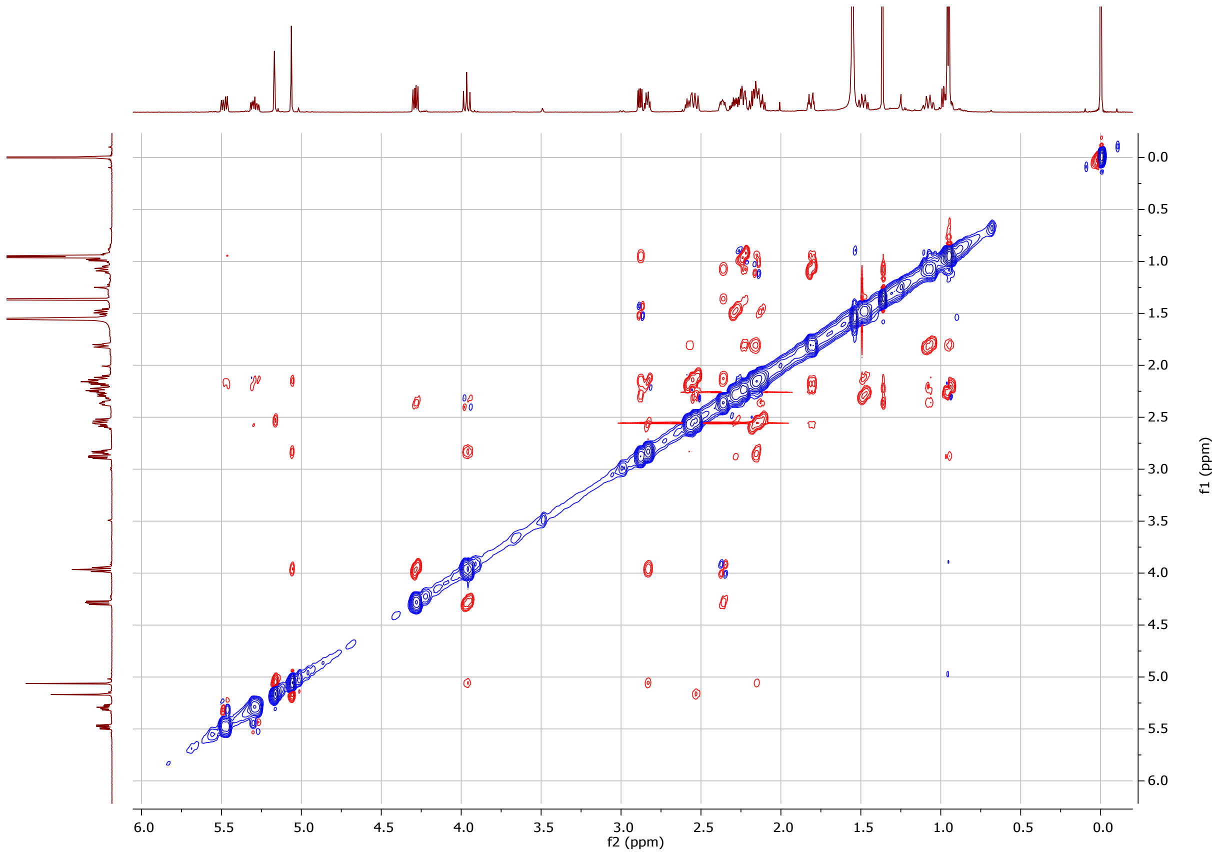

Figure S31: NOESY NMR spectrum of epoxycoraxeniolide A (**3**) in $CDCl_3$ (600 MHz).

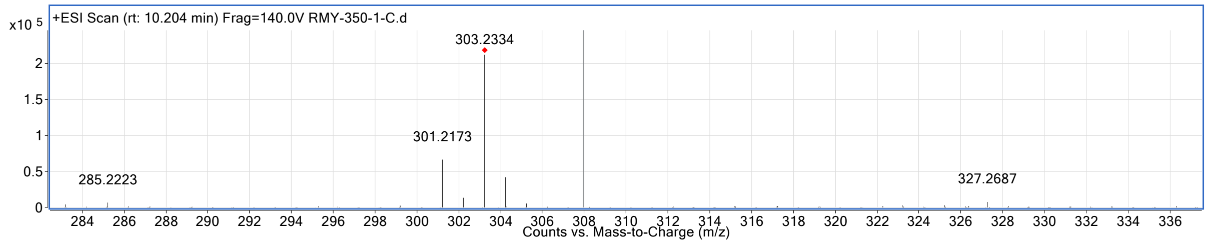

Figure S32: HRESIMS spectrum of coraxeniolide A (**4**)

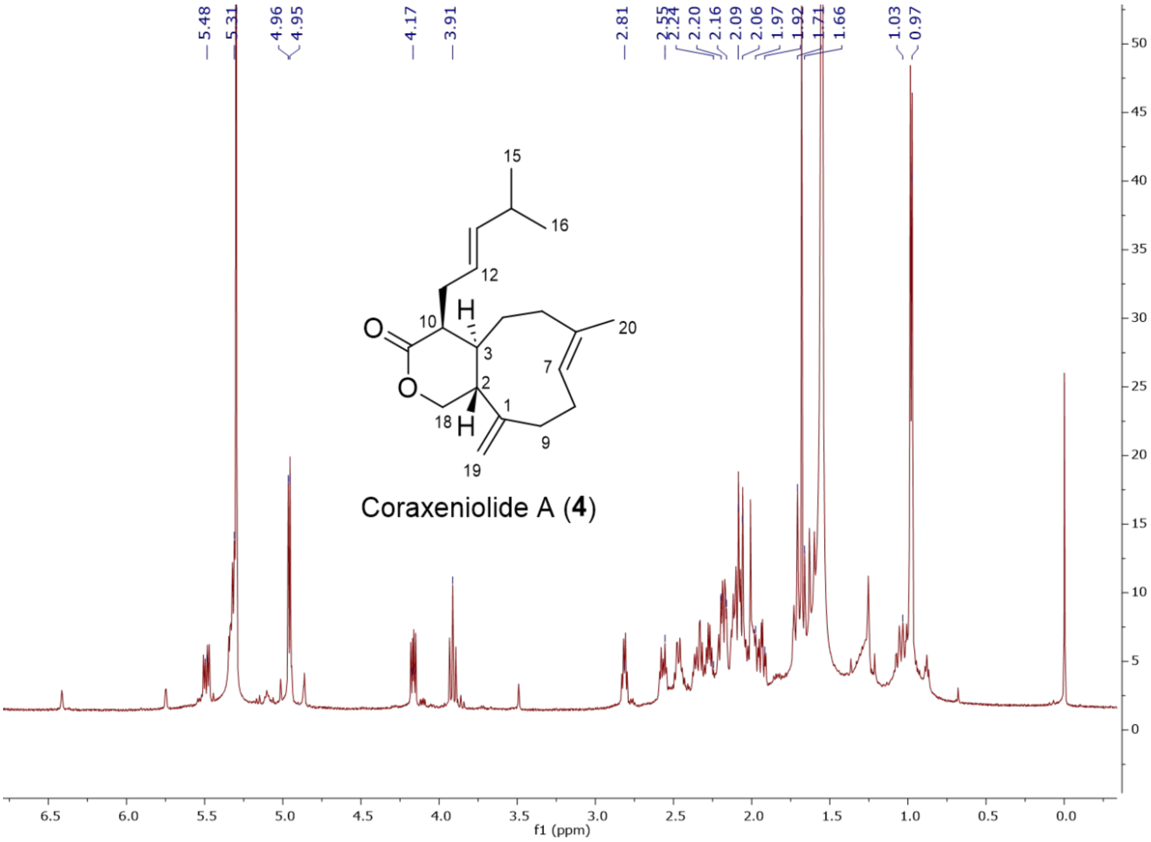

Figure S33: ^{1}H NMR spectrum coraxeniolide A (**4**) of in $CDCl_3$ (600 MHz).

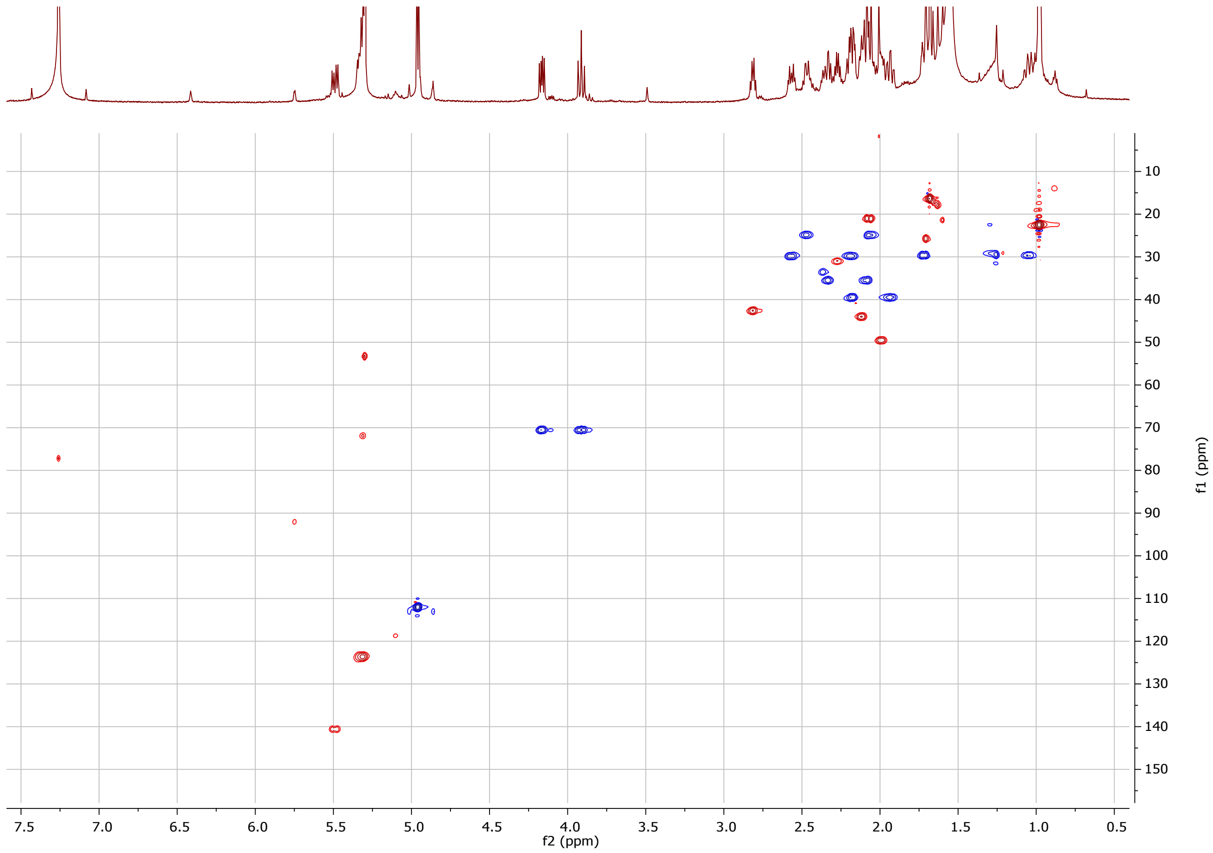

Figure S34: gHSQCAD spectrum coraxeniolide A (**4**) of in $CDCl_3$ (600 MHz).

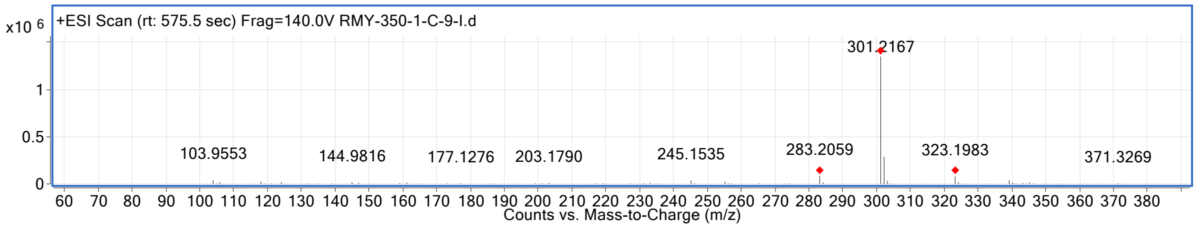

Figure S35: HRESIMS spectrum of coraxeniolide B (**5**).

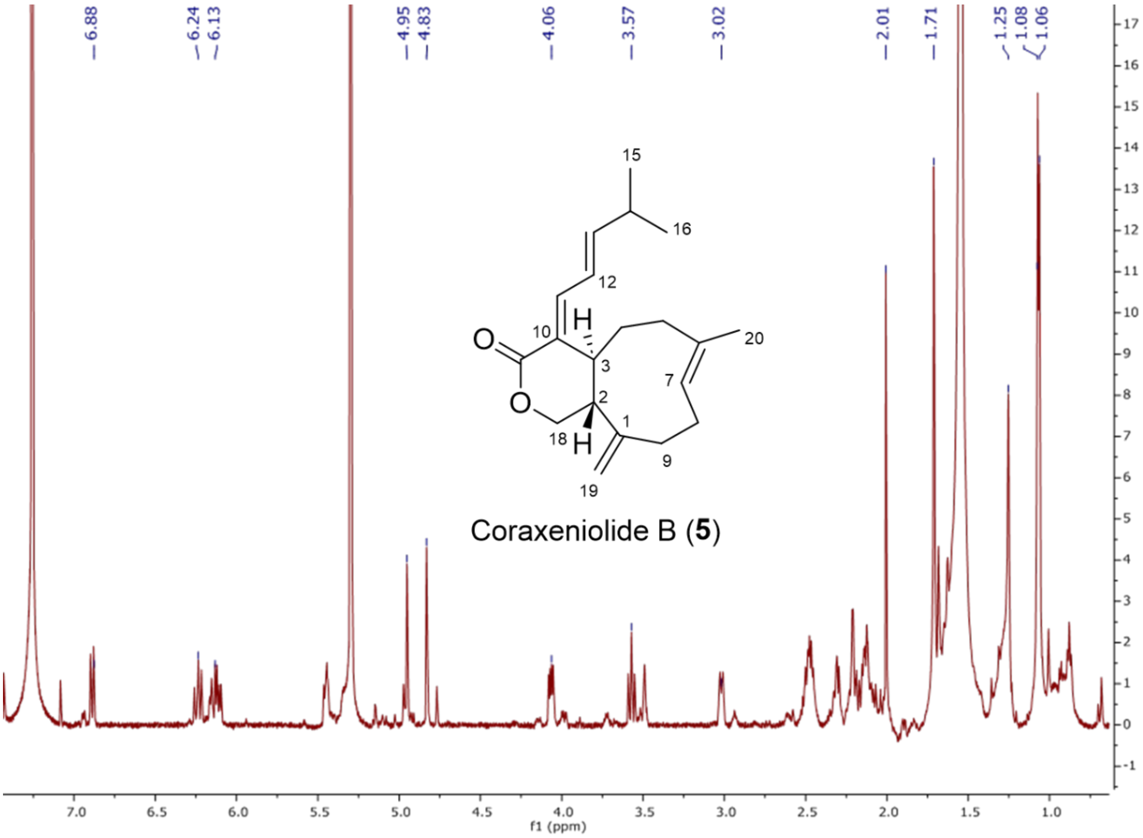

Figure S36: ^{1}H NMR spectrum coraxeniolide B (**5**) of in $CDCl_3$ (600 MHz).

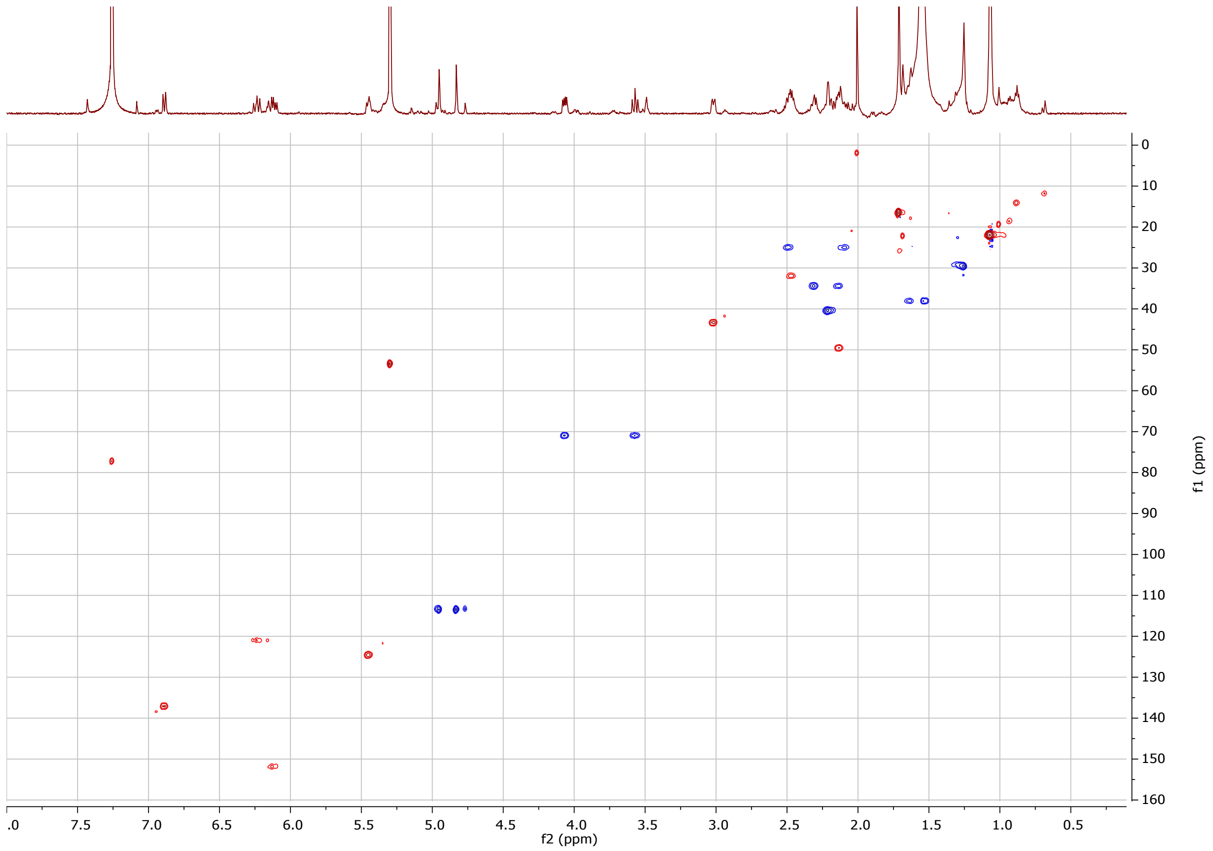

Figure S37: gHSQCAD NMR spectrum coraxeniolide B (**5**) of in $CDCl_3$ (600 MHz).

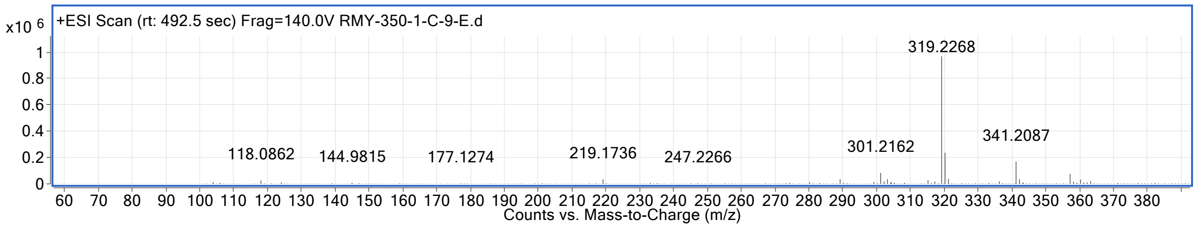

Figure S38: HRESIMS spectrum of acalycixeniolide F (**6**).

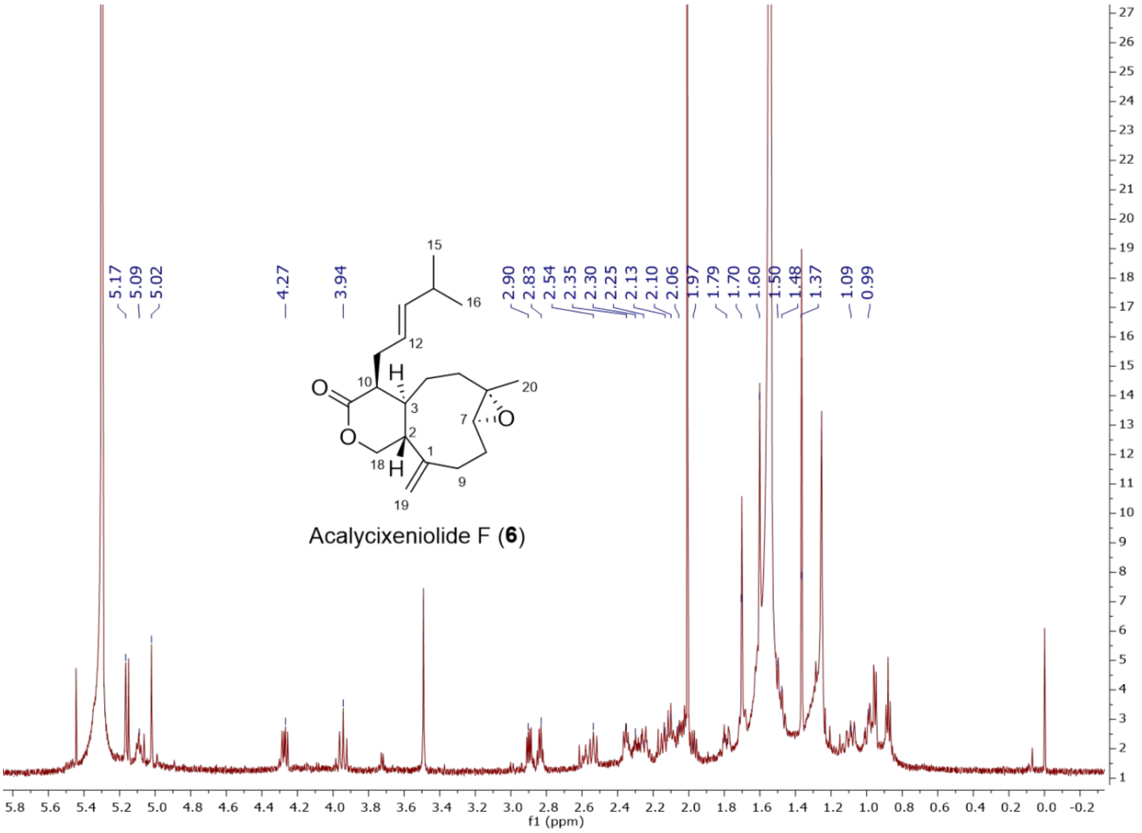

Figure S39: ^{1}H NMR spectrum of acalycixeniolide F (**6**) in $CDCl_3$ (600 MHz).

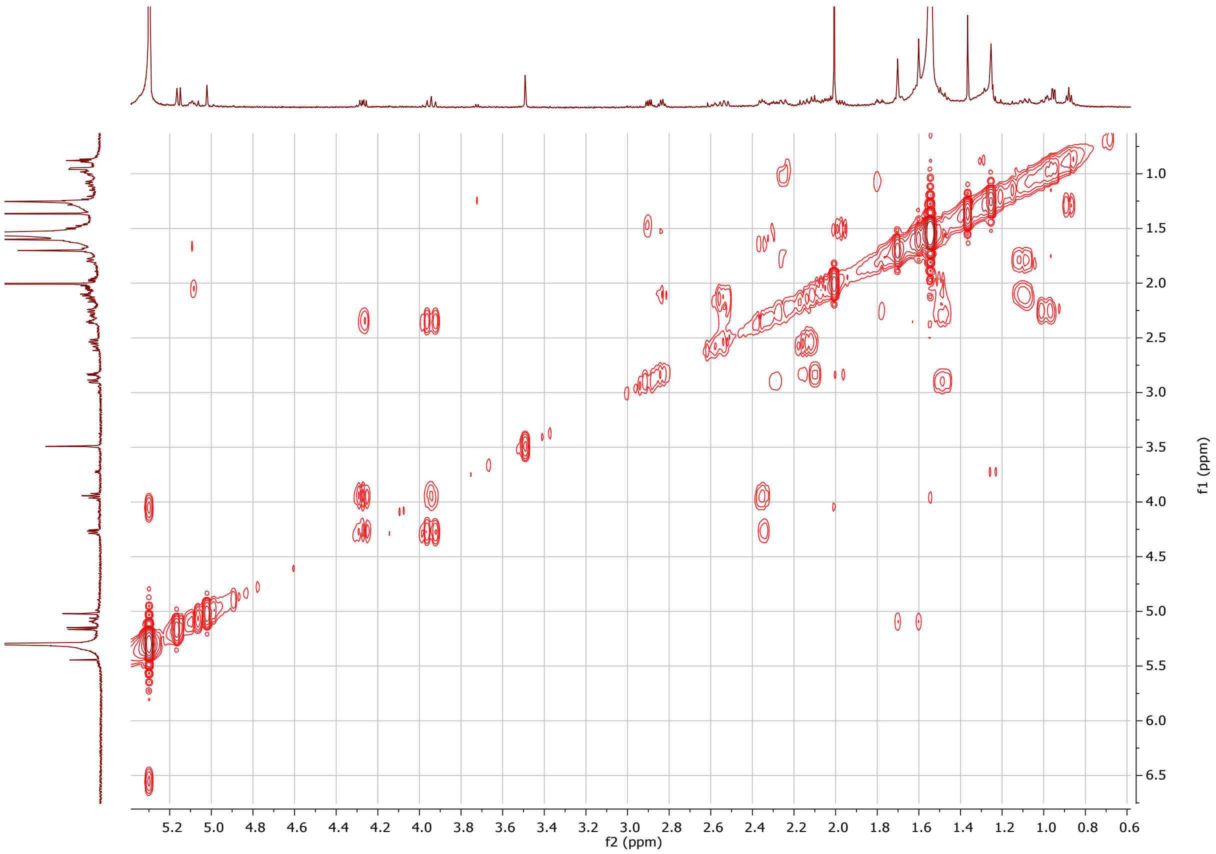

Figure S40: gCOSY NMR spectrum of acalycixeniolide F (**6**) in $CDCl_3$ (600 MHz).

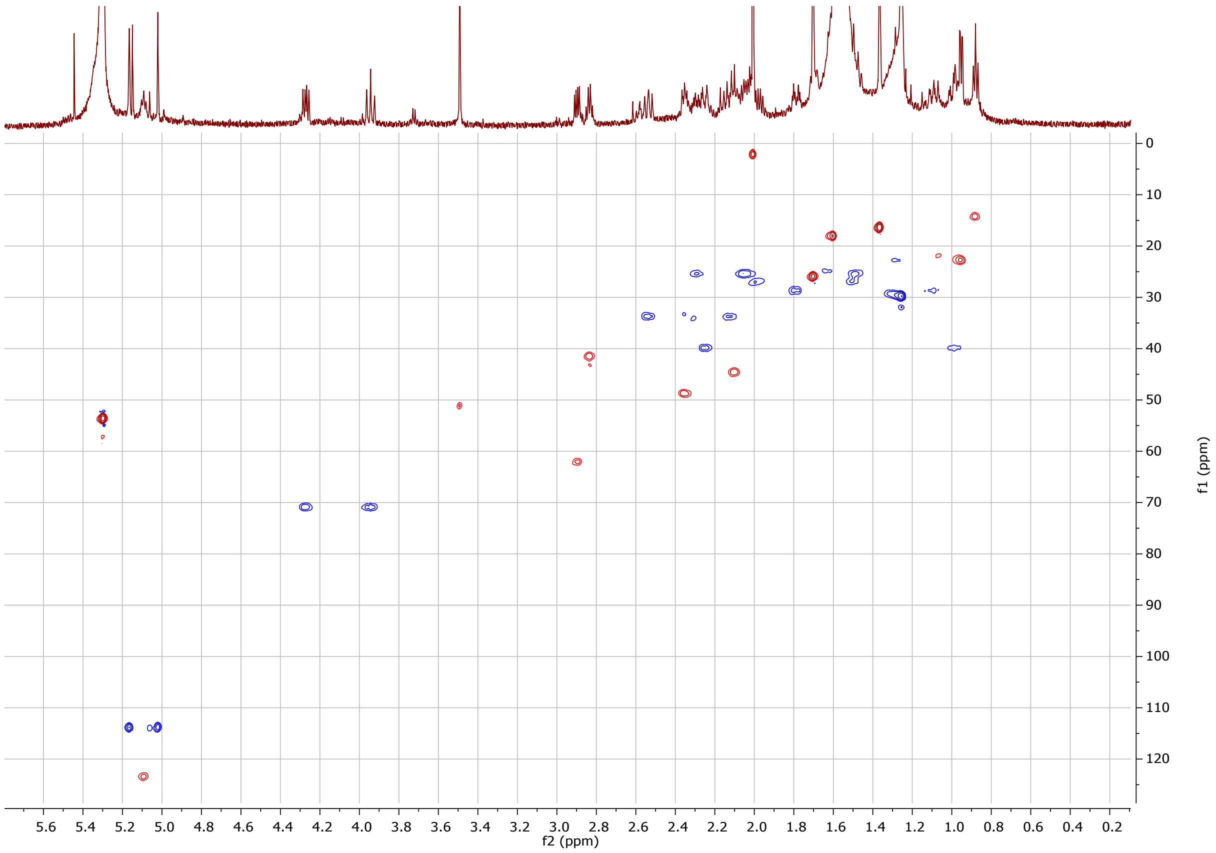

Figure S41: gHSQCAD NMR spectrum of acalycixeniolide F (**6**) in $CDCl_3$ (600 MHz).

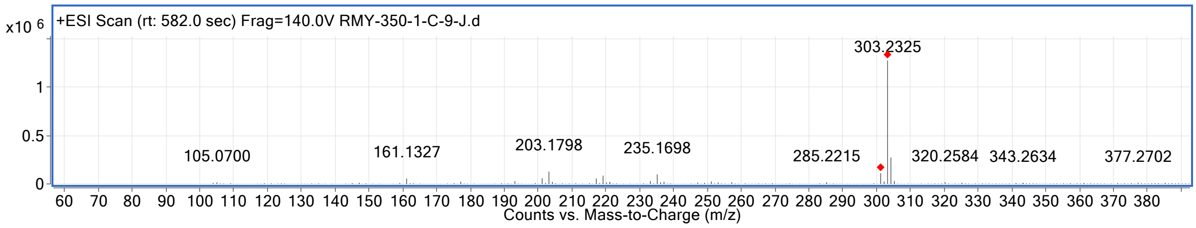

Figure S42: (+)HRESIMS spectrum acalycigorgin E (**7**).

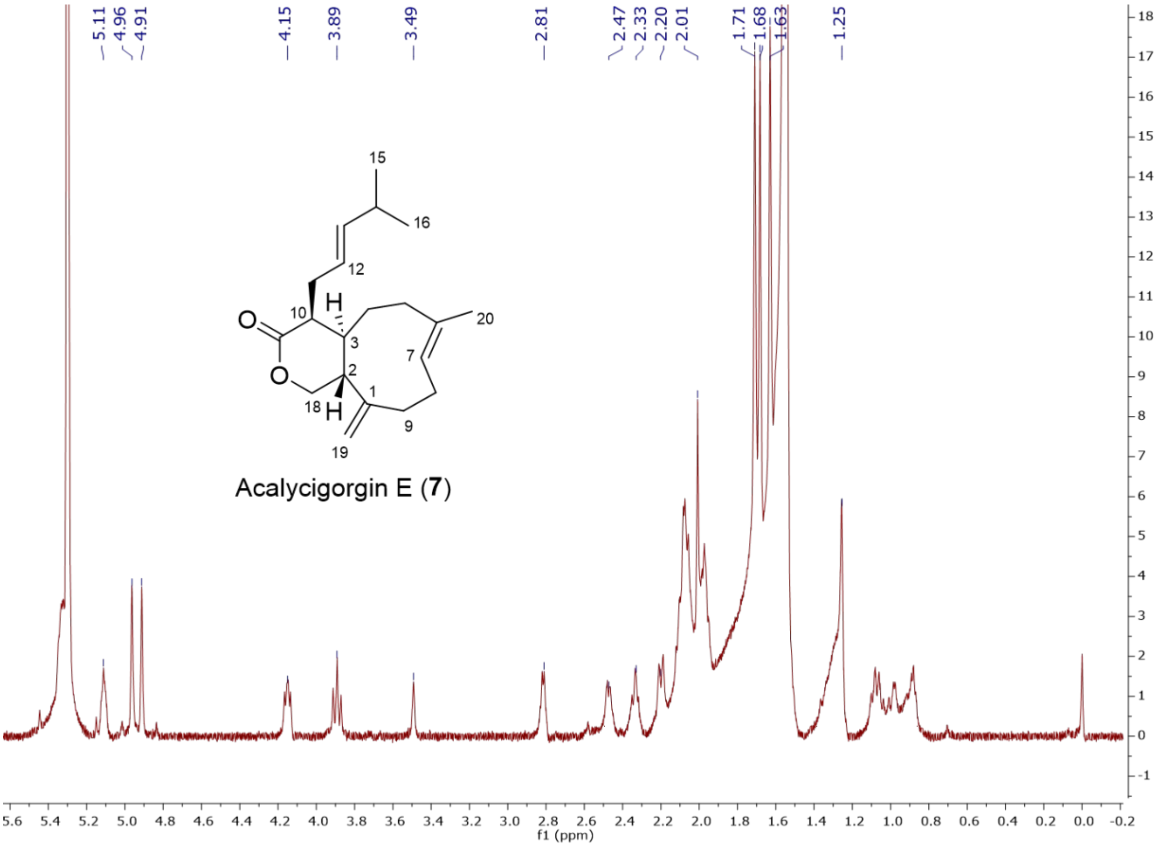

Figure S43:^{1}H NMR spectrum acalycigorgin E (**7**) of in $CDCl_3$ (600 MHz).

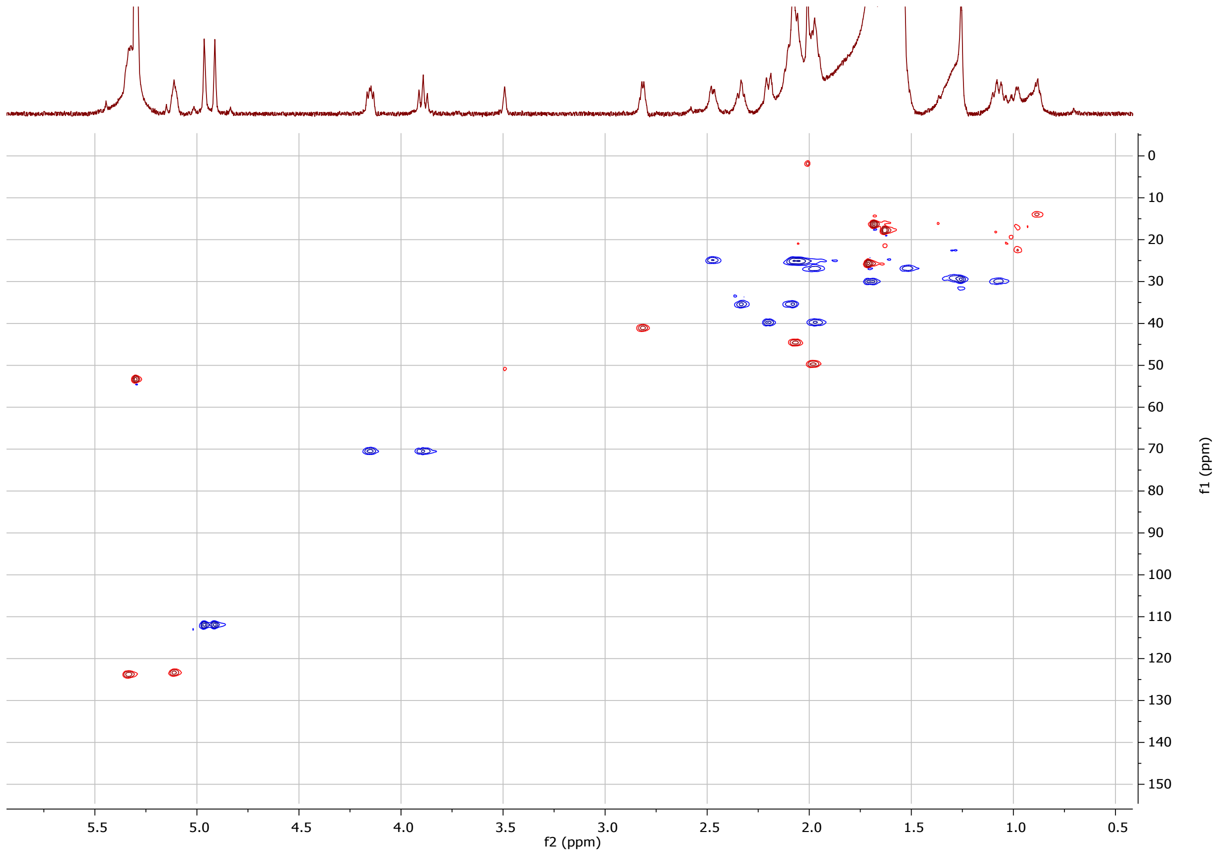

Figure S44: gHSQCAD NMR spectrum acalycigorgin E (**7**) of in $CDCl_3$ (600 MHz).

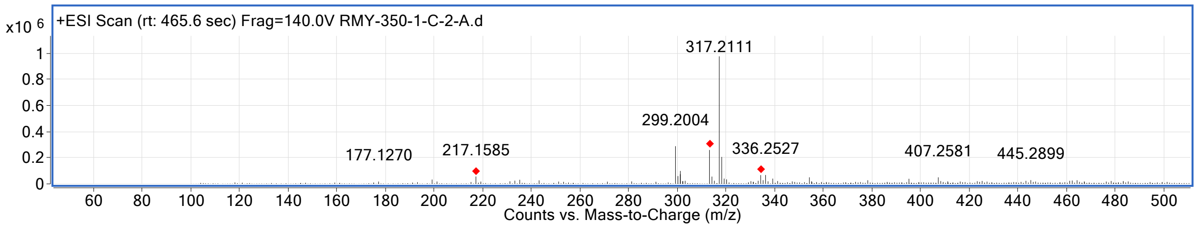

Figure S45:HRESIMS spectrum of 9-deoxyxeniolide A (**8**).

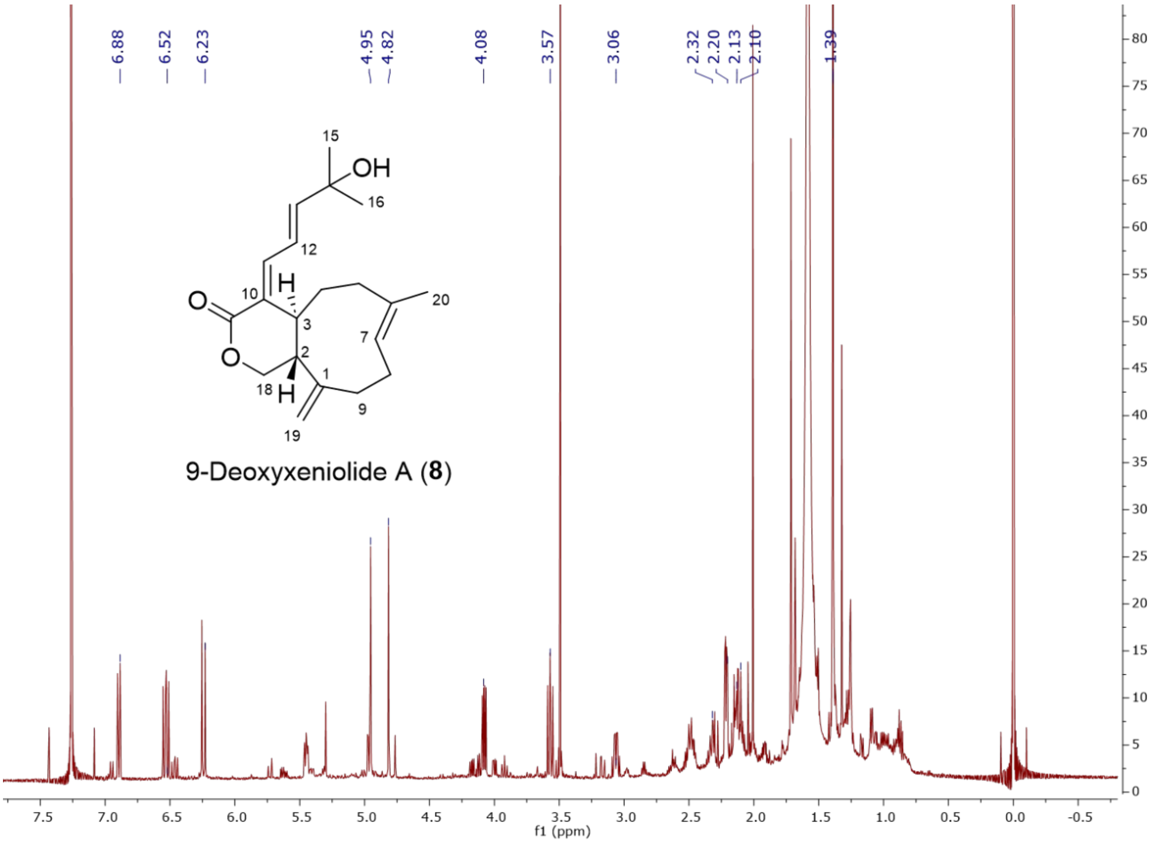

Figure S46: ^{1}H NMR spectrum 9-deoxyxeniolide A (**8**) of in $CDCl_3$ (600 MHz).

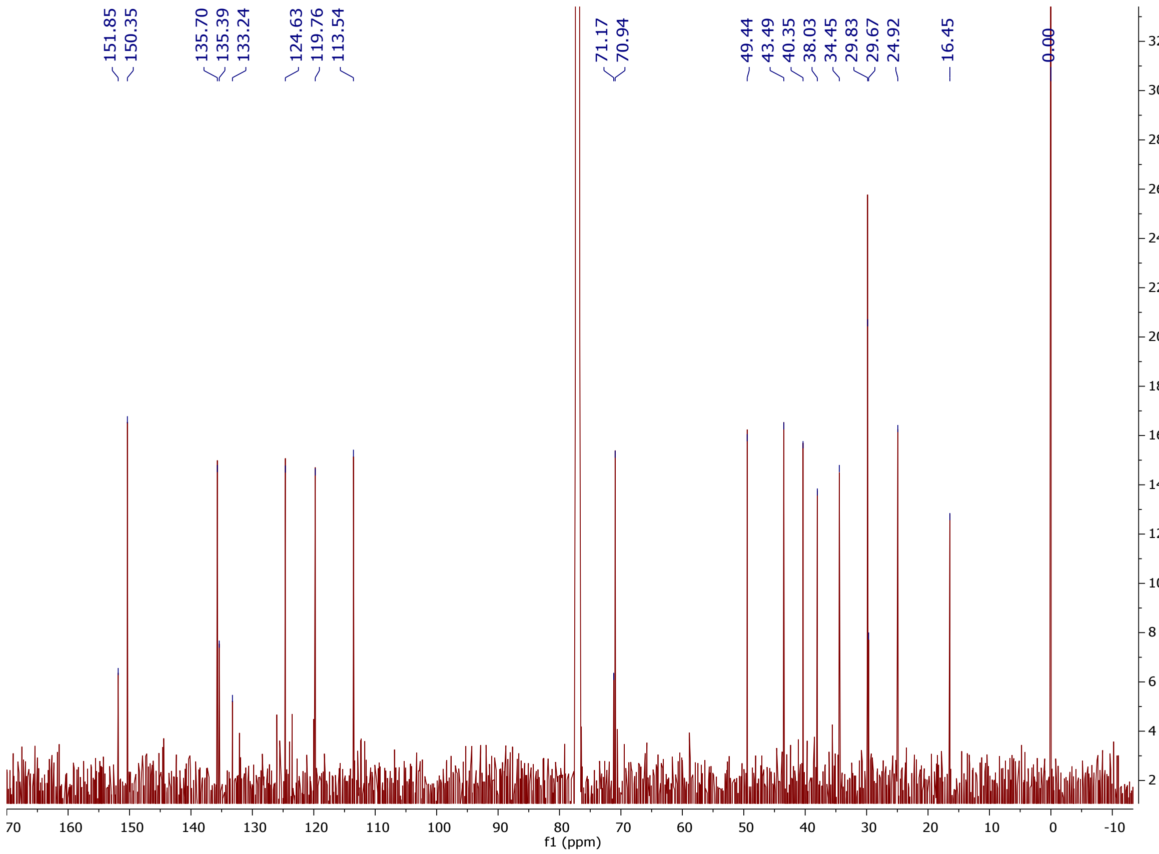

Figure S47: ^{13}C NMR spectrum of 9-deoxyxeniolide A (**8**) of in $CDCl_3$ (150 MHz).

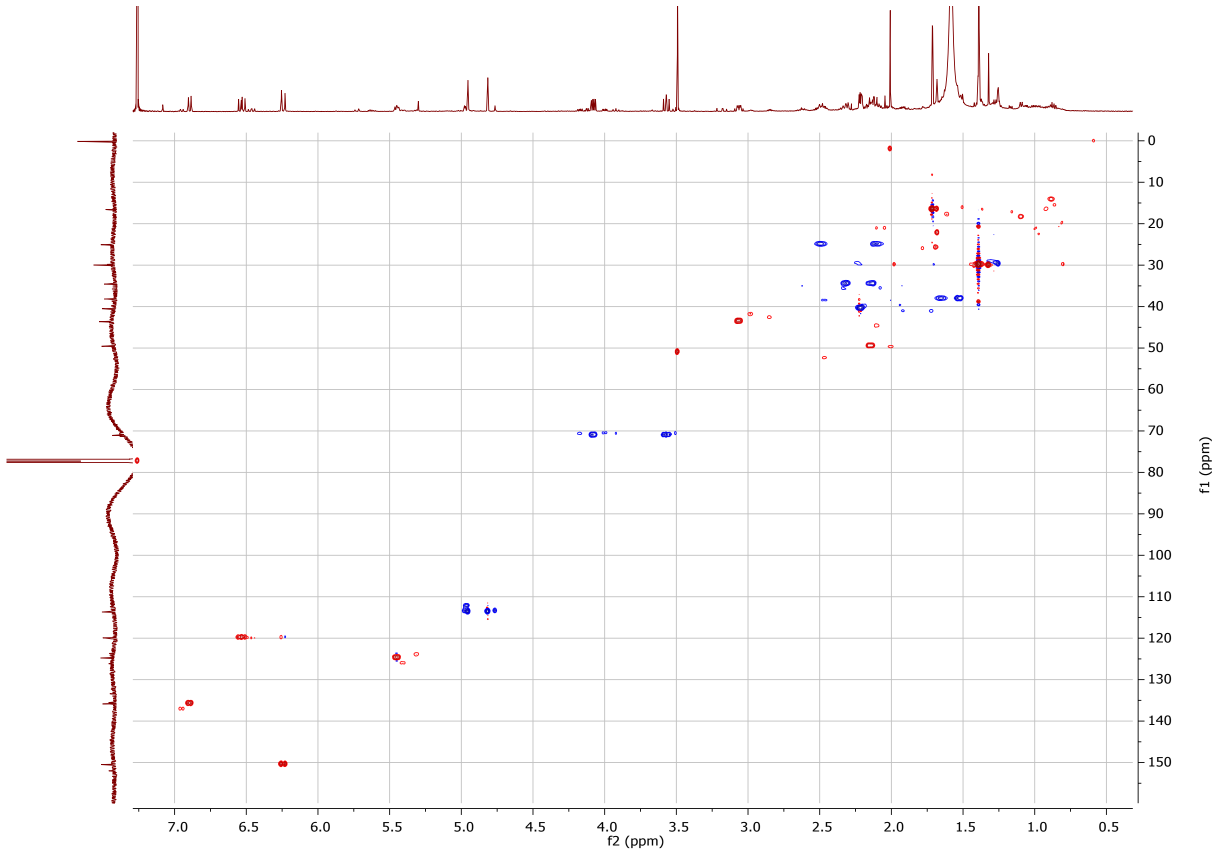

Figure S48: gHSQCAD NMR spectrum of 9-deoxyxeniolide A (**8**) of in $CDCl_3$ (600 MHz).

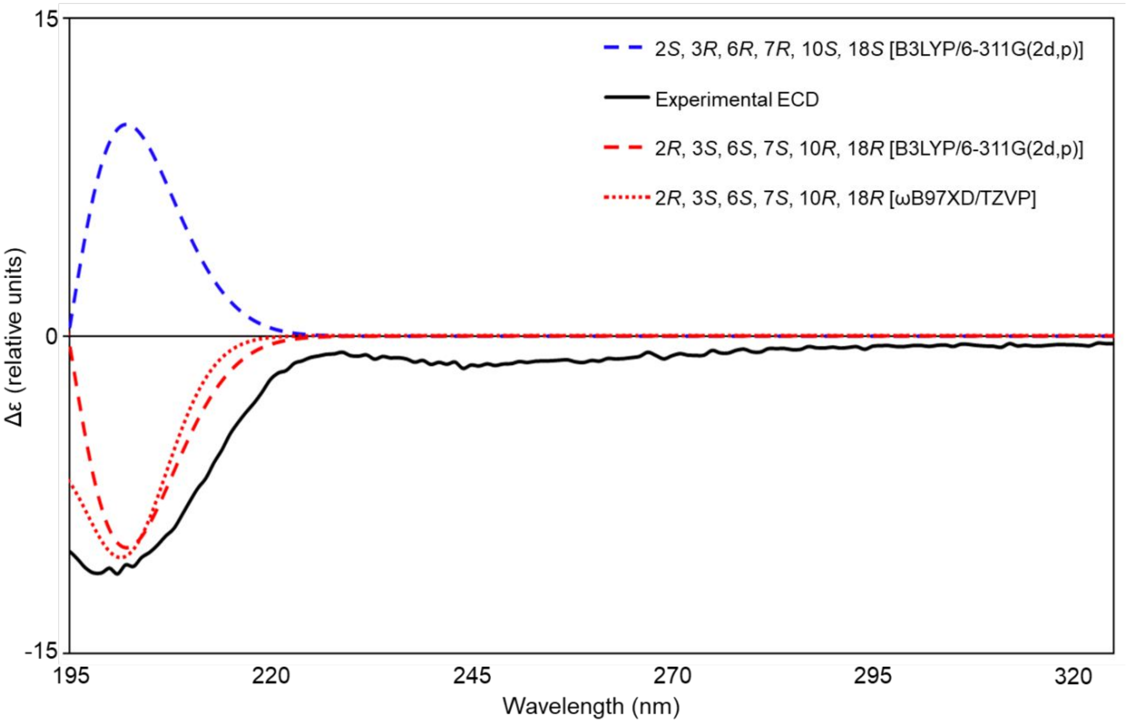

Figure S49: Calculated and experimental ECD spectra for epoxymiolenol (**2**) measured in acetonitrile.

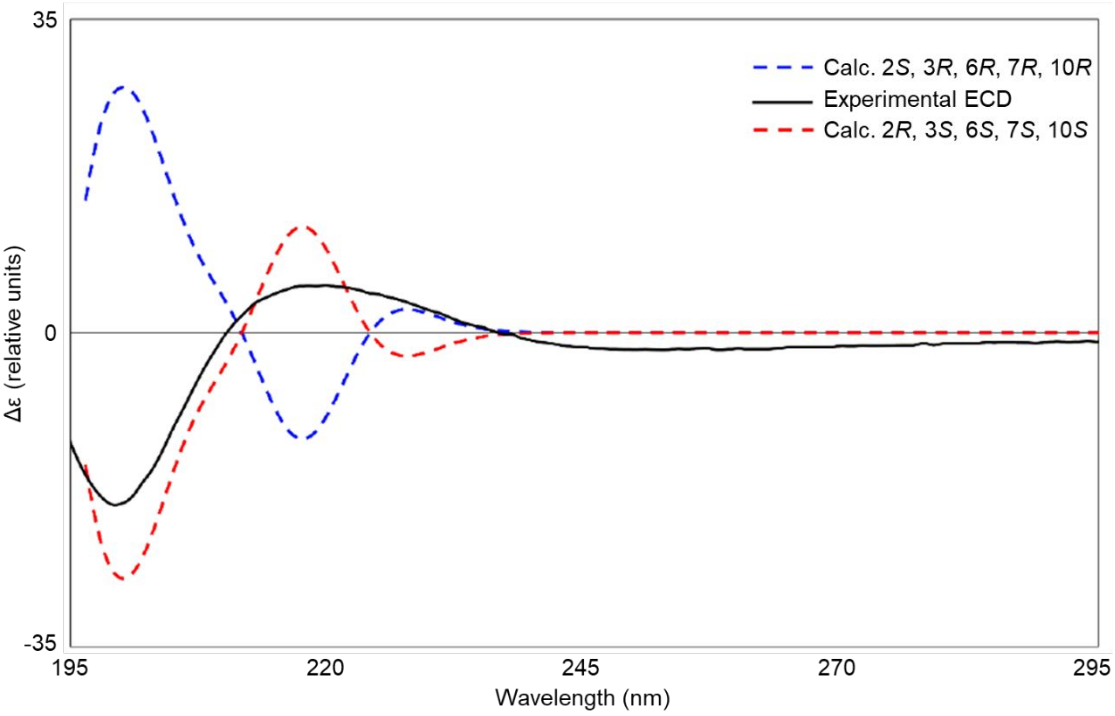

Figure S50 Calculated and experimental ECD spectra for epoxycoraxenolide (**3**) measured in acetonitrile.

Compound	%Activity NF54	%Activity Dd2
Coraxeniolide A (4)	8.79 (±20.7)	8.88 (±26.3)
Coraxeniolide B (5)	28.30 (±12.2)	23.88 (±37.7)
Acalycixeniolide F (6)	27.19 (±16.9)	44.38 (±32.1)
Acalycigogin E (7)	24.43 (±15.7)	23.79(±21.2)
Miolenol (1)	11.75 (±28.5)	28.15 (±23.6)
Epoxymiolenol (2)	53.57 (±30.3)	27.83 (±9.4)
Epoxycoraxeniolide A (3)	11.64 (±37.5)	26.66 (±22.7)
9-deoxyxeniolide A (8)	6.14 (±29.5)	57.09 (±4.9)
Chloroquine (CQ)	109.92 (±3.6)	100.22 (±3.6)
Dihydroartemisinin (DHA)	105.58(±3.4)	104.09 (±1.7)

Table S2: Antiplasmodial activities (% inhibition) of compounds **1-8,** tested against NF54 and Dd2.

Compound	EC_{50} NF54 (μM)	EC_{50} Dd2 (μM)
Coraxeniolide A	>5	>5
Coraxeniolide B	>5	>5
Acalycixeniolide F	>5	>5
Acalycigogin E	>5	>5
Miolenol	>5	>5
Epoxymiolenol B	>5	>5
Epoxycoraxeniolide A	>5	>5
9-deoxyxeniolide A	>5	>5

Table S3: Antiplasmodial activities (μM) of compounds **1-8,** tested against NF54 and Dd2.

NOESY Informed Distance Constraints			
Atoms	Forced Constraint (100%)	Distance (Å)	+/- (Å)
H-18/H-11	100	2.5	1.5
H-3/H-11	100	2.5	1.5
H-2/H-4	100	2.5	1.5
H-2/H-17	100	2.5	1.5
**Coupling Constant Informed Constraints**			
Atoms	Forced Constraint	Angle (°)	+/- (°)
H-2/H-3	100	90	30
H-2/H-17	100	90	30

Table S4: Conformer generation constraints informed by experimental NOESY correlations and coupling constant values.
